# Biodegradable Cu-based sonozymes for tumor-specific cuproptosis-enhanced sono-immunotherapy through activating cGAS-STING pathway and sensitizing immune checkpoint blockade

**DOI:** 10.1016/j.mtbio.2025.102397

**Published:** 2025-10-10

**Authors:** Yue Wu, Shangwei Xu, Jinming Cai, Jinyan Hu, Dengyu Pan, Bijiang Geng, Wen Gao, Yun Wu

**Affiliations:** a Department of Thoracic Surgery, Huadong Hospital, Fudan University, Shanghai, 200040, China; b Shanghai Key Laboratory of Clinical Geriatric Medicine, Huadong Hospital, Fudan University, Shanghai, 200040, China; c School of Environmental and Chemical Engineering, Shanghai University, Shanghai, 200444, China

**Keywords:** Cu_3_P, Cuproptosis, cGAS-STING, Sonodynamic therapy, Immunotherapy

## Abstract

Promoting the maturation of dendritic cells (DCs) is crucial for effectively activating the adaptive immune response. Nevertheless, DC maturation and subsequent antigen presentation were impeded by the immunosuppressive tumor microenvironments (TME). Herein, a cascade amplification strategy for the activation of antitumor immune response is reported for the first time based on a biodegradable Cu-based sonozyme, which integrates ROS-mediated immunogenic cell death (ICD), immune checkpoint blockade (ICB), cuproptosis, and cGAS-STING activation. An in-situ phosphating strategy was employed to synthesize Cu_3_P sonozymes from Cu_2_O nanocubes. Different from the pristine Cu_2_O, the obtained Cu_3_P nanocubes possess the TME-responsive degradation features, which can only degrade in tumor cells and cannot degrade in normal cells. By virtue of the TME-responsive degradation behaviors, a bioactive triterpenoid with reported antitumor activity (Cel) is then loaded on the surface of Cu_3_P nanocubes to achieve the tumor-specific drug release. In addition, Cu_3_P sonozymes exhibited higher sonodynamic and chemodynamic activities owing to the decreased bandgap and improved electronic structure. Immunosuppressive TME can be reversed by the cascade amplification ROS generation efficiency and tumor-specific cuproptosis. The tumor-specific cuproptosis can not only induce ICD, but also increase the expression of PD-LI in tumor cells and then sensitizes the ICB-mediated tumor therapy. Furthermore, Cu_3_P-mediated cuproptosis activates the cGAS-STING pathway and realizes the cascade amplification of antitumor immune response. This study introduces a new approach to achieve tumor-specific cuproptosis-enhanced sono-immunotherapy through activating cGAS-STING pathway and sensitizing ICB.

## Graphical abstract

Overall scheme to show the main synthesis procedures of Cu_3_P nanocubes through in-situ phosphating strategy for cuproptosis-enhanced sono-immunotherapy through activating cGAS-STING pathway and sensitizing immune checkpoint blockade.

**Figure:**
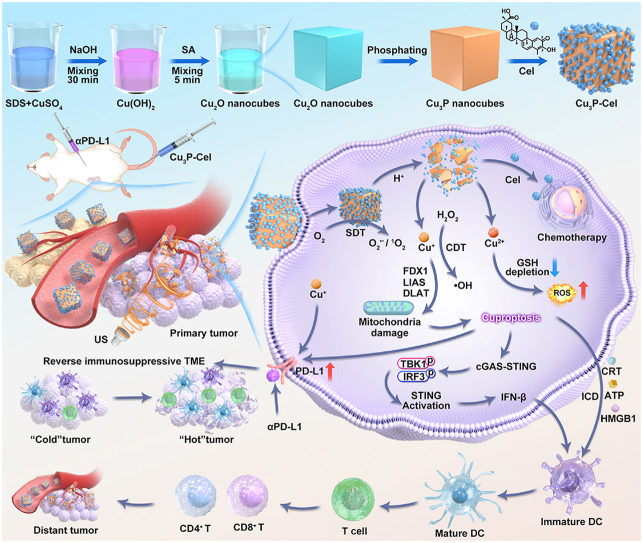

## 1 Introduction

Currently, the incidence and mortality rates of cancer are escalating, with lung cancer emerging as the most prevalent and lethal malignancy [[1], [2], [3]]. The current clinical treatments for lung cancer, including surgery, chemotherapy, and radiotherapy, have significant limitations: surgical trauma is large and cannot eliminate micro metastases; chemotherapy is highly toxic and prone to developing drug resistance; radiotherapy is limited to local areas and damages the surrounding normal tissues [[4], [5], [6], [7], [8], [9]]. In recent years, immune checkpoint inhibitors (ICIs), represented by lung cancer immunotherapy, have emerged as a significant breakthrough in the field [[10], [11], [12], [13]]. They have become a new line of treatment for patients with driver gene-negative, advanced, or locally advanced lung cancer, as well as those resistant to targeted therapies [[14], [15], [16]]. Unlike other anti-tumor approaches, immunotherapy focuses on activating the body's own immune system to create a less favorable “soil” for tumor growth and indirectly eliminate cancer cells [[17], [18], [19]]. Programmed death-ligand 1 (PD-L1) inhibitors are representative immune checkpoint inhibitors used in lung cancer treatment [[20], [21], [22]]. Nevertheless, a considerable number of patients continue to show inadequate response to ICB because of low PD-L1 expression [[23], [24], [25], [26]]. Furthermore, patients undergoing αPD-L1 therapy commonly face challenges such as drug resistance or immune-related adverse events [[27], [28], [29]]. While enhanced anti-PD-L1 therapy has shown efficacy in treating lung cancer in combination with different therapeutic strategies, such as sonodynamic therapy (SDT), chemodynamic therapy (CDT), and phototherapy [[30], [31], [32], [33], [34], [35], [36], [37]], there is still a requirement for novel strategies that can directly increase the level of tumor-infiltrating lymphocytes in the TME to transform a “cold” tumor into a “hot” tumor and improve the potential responses to ICB.

Recently, the emergence of programmed cell death mechanisms such as ferroptosis, pyroptosis, and cuproptosis provides opportunities for novel therapy strategies to overcome resistance to apoptosis and activate immunotherapy [[38], [39], [40], [41], [42]]. Cuproptosis represents a unique form of copper-induced cell death [[43], [44], [45]]. The mechanism involves an overabundance of copper ions (Cu^+^) hindering mitochondrial metabolic activity [[46], [47], [48]]. Furthermore, cuproptosis can induce immunogenic cell death (ICD) in tumor cells, releasing damage-related molecular patterns, promoting the maturation of dendritic cells (DCs) and the infiltration of cytotoxic T lymphocyte [[49], [50], [51], [52], [53]]. In addition to the induction of ICD, cuproptosis also has the ability to increase the expression of PD-L1 in tumor cells and activate the cGAS-STING pathway [[52], [53], [54]]. Many previous reports revealed that the activation of cGAS-STING pathway has been demonstrated to play important roles in enhancing DC maturation [[55], [56], [57], [58]]. Nonetheless, the exploration of cuproptosis is still in its early phases and needs to confront the following challenges. A key factor that impacts the efficacy of tumor therapy is enhancing the accumulation of copper ions within tumor cells, as indicated by the analysis of cuproptosis mechanisms [59,60]. Regular dosing is often necessary to increase copper ion levels in tumor tissues for maximum therapeutic effectiveness [61,62]. Yet, cuproptosis-mediated tumor therapy still lacks high selectivity or intelligence, as the accumulation of copper ions in normal tissues could lead to potential safety risks [63,64]. Consequently, the development of a stimulus-responsive nanoplatform tailored as a copper ionophore to induce tumor-specific cuproptosis is imperative. Moreover, glutathione (GSH) is able to inhibit the binding of Cu ions to the lipoylated components of the tricarboxylic acid (TCA) cycle, which could lessen the therapeutic effectiveness of cuproptosis [65,66]. Thus, in addition to achieving tumor-specific cuproptosis, the next hurdle to overcome is the depletion of GSH through modulation of the TME.

The narrow bandgap and presence of Cu ^+^ endow semiconductor oxide Cu_2_O with the ability to serve as a sonosensitizer and nanozyme [67]. Furthermore, Cu_2_O possesses excellent degradation features, which endowed Cu_2_O with the cuproptosis induction capability [68]. On this basis, Cu_2_O-based nanomaterials are expected to combine cuproptosis-mediated tumor therapy with SDT and CDT, then activating a powerful adaptive immune response. Unfortunately, the feature of Cu_2_O being easily oxidized and degraded is a double-edged sword. Degradation can occur in acidic TME as well as under normal physiological conditions, leading to the risk of cuproptosis in normal cells [49,68]. Furthermore, the fast electron-hole pair recombination in Cu_2_O-based nanomaterials hinders their potential application as a sonosensitizer in SDT [69,70].

**Scheme 1:**
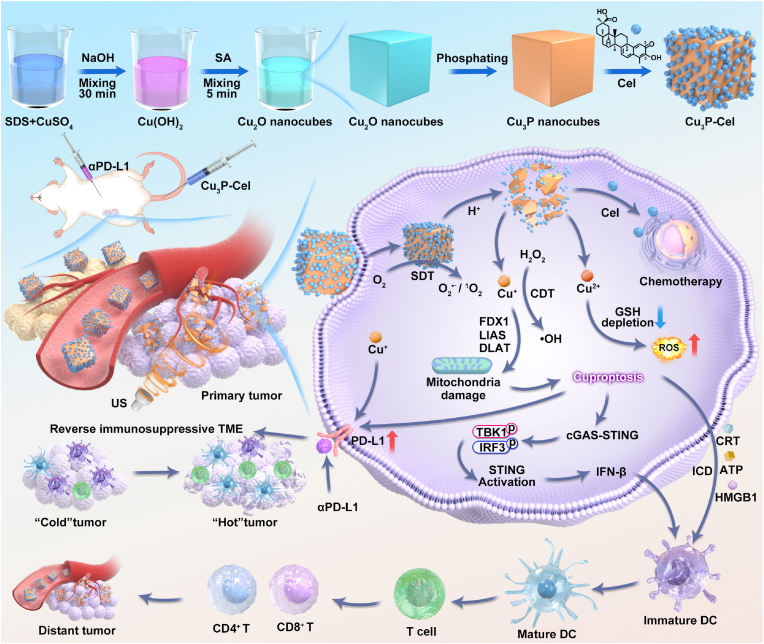
Schematic illustration of the preparation of Cu_3_P nanocubes through in-situ phosphating strategy for cuproptosis-enhanced sono-immunotherapy through activating cGAS-STING pathway and sensitizing immune checkpoint blockade.

In this work, we report for the first time the in-situ phosphating strategy of Cu_2_O nanocubes to regulate the degradation behavior of Cu_2_O nanocubes. Compared with the Cu_2_O nanocubes, the obtained Cu_3_P nanocubes can only degrade in tumor cells and cannot degrade in normal cells, endowing the TME-responsive degradation feature with Cu_3_P nanocubes. The potential clinical translation advantages of Cu_3_P can be highlighted through an examination of the tumor-specific degradation features of Cu_3_P nanocubes. First, the prepared Cu_3_P nanocubes exhibited the narrower bandgap compared with the Cu_2_O nanocubes, revealing the higher sonodynamic activity. Second, the Cu ions released specifically to the tumor can initiate the cascade amplification of ROS generation, causing tumor-specific cuproptosis and resulting in significant cellular destruction that triggers ICD effects. Third, the Cu_3_P-induced cuproptosis effect not only increases the expression of PD-L1 in tumor cells and then sensitizes the ICB-mediated tumor therapy, but also activates the cGAS-STING pathway and realizes the cascade amplification of antitumor immune response. Fourth, the TME-responsive degradation features make Cu_3_P nanocubes an ideal drug loading nanoplatform, which achieves tumor-specific drug release by loading a bioactive triterpenoid with reported antitumor activity (Cel) for lung cancer, further enhancing the antitumor effectiveness. Cu_3_P-Cel-αPD-L1-mediated cuproptosis-augmented sonocatalytic-immunotherapy have been shown to have significant antitumor effects in eradicating primary tumors and suppressing the growth of distant tumors (Scheme 1). Moreover, different form various non-biodegradable sonozymes, Cu_3_P without obvious long-term toxicity exhibited good potential for in vivo applications because Cu_3_P nanocubes have the ability to break down into Cu and P ions that are swiftly removed by the body.

## 2 Results and discussion

**Fig. 1:**
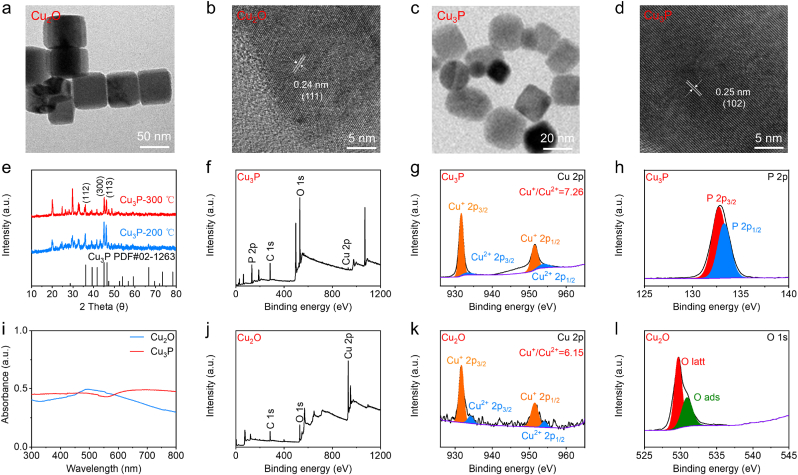
(a–d) The morphology, size, lattice structure of Cu_2_O and Cu_3_P. (e–l) The crystal structure, elementary composition, and optical properties of Cu_2_O and Cu_3_P.

Cu_3_P nanocubes were synthesized from Cu_2_O nanocubes through the phosphating process, which involved the calcination process in a tube furnace. The cubic morphology of Cu_2_O nanocubes (approximately 50 nm) can be found in the TEM image presented in Fig. 1a. The high-resolution TEM image of Cu_2_O nanocubes revealed a distinct lattice fringe, indicating that the 0.24 nm lattice fringe corresponded to the (111) plane (Fig. 1b). This image also showed the good crystallization of Cu_2_O nanocubes. Based on this situation, we then synthesized Cu_3_P nanocubes from Cu_2_O nanocubes through calcination process at two different temperatures. Compared with the calcination temperature of 200 °C, the Cu_3_P prepared at the calcination temperature of 300 °C possessed the higher crystallization (Fig. 1e). The good crystallinity and 0.25 nm lattice fringe were verified by the high-resolution TEM image of Cu_3_P nanocubes (Fig. 1d). TEM image of Cu_3_P nanocubes revealed that their size was approximately 25 nm (Fig. 1c). The size measurement results of Cu_3_P nanocubes and Cu_2_O nanocubes were further verified by the DLS results (Fig. S1).

We then investigated the chemical structure of Cu_3_P nanocubes and Cu_2_O nanocubes. As depicted in Fig. 1j, the Cu and O elements can be found in the survey XPS spectrum of Cu_2_O nanocubes. The abundant Cu^+^ and a small number of Cu^2+^ can be detected in the high-resolution Cu 2p spectrum of Cu_2_O (Fig. 1k), which could be attributed to the easy oxidation features of Cu_2_O nanocubes. Lattice oxygen and absorbed oxygen were observed in the high-resolution O 1s spectrum of Cu_2_O nanocubes (Fig. 1l). The appearance of absorbed oxygen could also be ascribed to the easy oxidation features of Cu_2_O nanocubes, which easily absorbed moisture from the air. For comparison, the P and Cu elements can be detected in the survey XPS spectrum of Cu_3_P nanocubes (Fig. 1f). The appearance of P element in Cu_3_P demonstrated successful preparation of Cu_3_P nanoparticles. The high-resolution P 2p spectrum of Cu_3_P exhibited two peaks, corresponding to the P 2p_3/2_ and P 2p_1/2_ of Cu_3_P (Fig. 1h). Similar to Cu_2_O nanocubes, Cu_3_P nanocubes also contained Cu^+^ and Cu^2+^ (Fig. 1g). However, the amount of Cu^2+^ in Cu_3_P nanocubes was significantly lower than that in Cu_2_O nanocubes, manifesting that Cu_3_P was not easily oxidized. We also measured the surface charge characteristics of these two samples. As presented in Fig. S2, no significant change of Zeta potential of these two samples can be detected. The excellent colloidal stability of Cu_3_P nanocubes can be demonstrated in Fig. S3a and b, which revealed no color or absorption changes of solution after storing for different times. Even in low-temperature (4 °C) and normal animal body temperature (37 °C) environments, Cu_3_P nanocubes exhibited excellent colloidal stability, which can be seen from the fact that its particle size and zeta potential did not change significantly (Fig. S3c and d). The higher absorbance of Cu_3_P nanocubes at NIR region can be detected in the absorption spectrum compared with Cu_2_O nanocubes (Fig. 1i), indicating that Cu_3_P nanocubes possessed a lower bandgap structure.

**Fig. 2:**
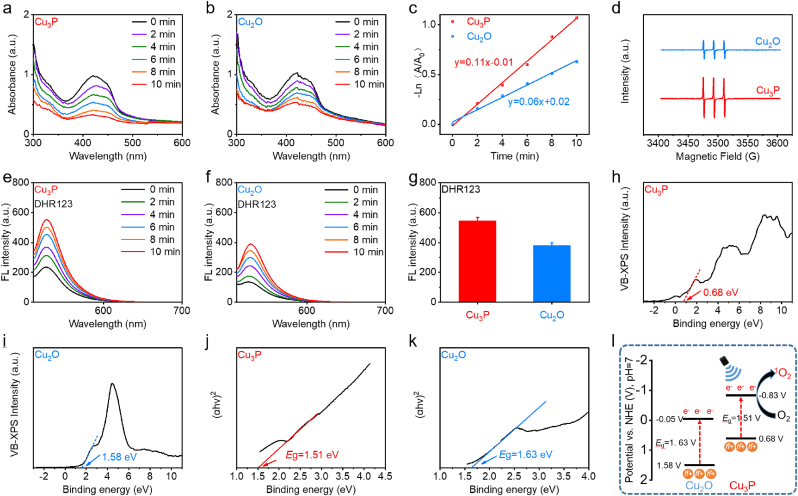
(a–g) Evaluation and comparison of ROS (^1^O_2_ and O_2_^•^ˉ) generation capability of these two samples using absorption spectrum, ESR, and fluorescence spectrum under US irradiation (50 kHz, 1.0 W/cm^2^, 50 % duty cycle). (h–k) Measurements of the bandgap structures of Cu_2_O and Cu_3_P. (l) Schematic illustration of the bandgap structure of Cu_2_O and Cu_3_P. Data are presented as the mean ± SD. (n = 3).

Upon demonstrating the preparation of Cu_2_O and Cu_3_P, the amplified US-activated ROS generation of Cu_3_P was then assessed. DPBF can simultaneously detect ^1^O_2_ and O_2_^•^ˉ through absorption spectrum. As illustrated in Fig. 2a and b, the peak of DPBF exhibited gradual decrease in Cu_3_P and Cu_2_O as prolonging the US irradiation time. For comparison, Cu_3_P exhibited the lower DPBF absorbance after US irradiation for 10 min, suggesting the higher sonodynamic activity of Cu_3_P. To more accurately compare the sonodynamic activity, we calculated the rate constant of ROS generation in these two groups. The higher rate constant of ROS generation can be detected in the Cu_3_P group compared with the Cu_2_O group at the same irradiation conditions (Fig. 2c). Because DPBF cannot distinguish the type of ROS, we then utilized TEMP as the ^1^O_2_ probe to detect and compare the ^1^O_2_ generation efficiency of Cu_3_P and Cu_2_O through ESR spectrum. Fig. 2d exhibited that the stronger ESR signal of ^1^O_2_ was detected in the Cu_3_P group compared with the Cu_2_O group, manifesting that the phosphating of Cu_2_O could improve the sonodynamic activity of Cu_2_O. The O_2_^•^ˉ was then measured by DHR123. As illustrated in Fig. 2e–g, the higher fluorescence intensity of DHR123 can be detected in the Cu_3_P group compared with the Cu_2_O group, which was consistent with the above results. These results also demonstrated that the sonocatalytic of Cu_3_P involved both energy transfer and electron transfer process, which can produce both ^1^O_2_ and O_2_^•^ˉ.

We then explored the energy band structure of these two kinds of semiconductor sonosensitizers to clarify the sonodynamic mechanism. To illustrate the mechanism of enhanced sonodynamic activity, the bandgap value and reduction potential of semiconductor sonosensitizers should be measured. First, we can calculate the bandgap value of these two semiconductor sonosensitizers through their absorption curves. As illustrated in Fig. 2j and k, the values of bandgap in Cu_2_O and Cu_3_P were approximately 1.63 and 1.51 eV, respectively. It is well known that the lower bandgap value could endow semiconductor sonosensitizers with higher sonodynamic activity. Hence, Cu_3_P with lower bandgap value revealed higher US-activated ROS production when compared with Cu_2_O. In addition to bandgap value, the reduction potential of O_2_ to O_2_^•^ˉ was also considered as the influence factor of the sonodynamic property of semiconductor sonosensitizers. This value can be obtained by measuring the values of valence band (VB) potential. Fig. 2h, i exhibited that the values of VB potential for Cu_3_P and Cu_2_O were about 0.68 and 1.58 eV, respectively. Consequently, we can calculate the values of conduction band (CB) potential, which was −0.83 and −0.05 eV, respectively (Fig. 2l). Therefore, the enhanced sonodynamic activity of Cu_3_P could be ascribed to the more negative conduction band potential (*E*_CB_) and narrower bandgap (*E*_g_) of Cu_3_P compared with Cu_2_O.

**Fig. 3:**
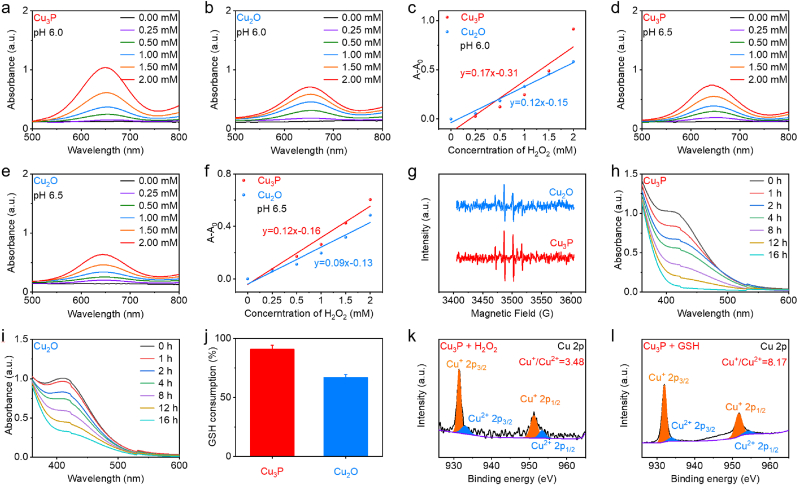
(a–f) The increase extent of the peak of TMB with the addition of Cu_2_O and Cu_3_P at pH 6.0 and 6.5. (g) ESR peaks in different groups with the addition of DMPO. (h–j) The reduction extent of the peak of DTNB with the addition of Cu_2_O and Cu_3_P. (k, l) The change of valence state of Cu in Cu_3_P after the addition of H_2_O_2_ or GSH. Data are presented as the mean ± SD. (n = 3).

Having demonstrated that the phosphating process would influence the sonodynamic activity of Cu_2_O, we then investigated whether the chemodynamic activity and GSH depletion performance of Cu_2_O could be enhanced after the phosphating process. The higher the H_2_O_2_ concentration, the more TMB was absorbed at 652 nm (Fig. 3a and b), showing the production of •OH with Cu_3_P and Cu_2_O. Furthermore, a significant disparity was observed in the Cu_3_P group (0.17 min^−1^) and the Cu_2_O group (0.12 min^−1^), as depicted in Fig. 3c. After the phosphating of Cu_2_O, the sonodynamic activity of the Cu_3_P was enhanced through the decreased bandgap and more negative *E*_CB_. At pH 6.5, Fig. 3d–f demonstrated that Cu_3_P and Cu_2_O are capable of producing •OH. The •OH production efficiency of Cu_3_P at pH 6.5 was greater than that of the Cu_2_O nanocubes, just like at pH 6.0. Additionally, the production of •OH was observed to be influenced by pH in both Cu_3_P and Cu_2_O, which demonstrated that a higher rate constant was observed at pH 6.0 in comparison to pH 6.5 (Fig. 3c–f). No •OH generation was observed in Cu_3_P and Cu_2_O at pH 7.4 (Fig. S4). The enhanced Fenton-like reaction activity of Cu_3_P compared with Cu_2_O can be also demonstrated by the ESR spectrum (Fig. 3g).

We further investigated whether Cu_3_P and Cu_2_O could deplete GSH due to the presence of Cu^2+^ in Cu_3_P and Cu_2_O. The presence of GSH was detected in the samples containing Cu_3_P and Cu_2_O through the use of DTNB at varying incubation time points. Following a 16-h incubation, Fig. 3h revealed a distinct decrease of the DTNB peak in the Cu_3_P solution. Furthermore, Cu_2_O demonstrated a GSH depletion capability lower than that of Cu_3_P (Fig. 3i and j), which can be attributed to the low catalytic activity of Cu_2_O. We then investigated the catalytic mechanism of Cu_3_P through measuring the XPS spectrum of Cu_3_P after reacting with H_2_O_2_ or GSH. As depicted in Fig. 3k, the ratio of Cu^2+^ increased in Cu_3_P + H_2_O_2_ after reacting with H_2_O_2_, illustrating that the Cu^+^ was oxidated to Cu^2+^ during the Fenton-like reaction. Fig. 3l exhibited that the ratio of Cu ^+^ increased in Cu_3_P + GSH after reacting with GSH, suggesting that the Cu^2+^ was reduced to Cu^+^ during the GSH depletion process. The cycle between Cu^+^ and Cu^2+^ would endow Cu_3_P with excellent chemodynamic activity and GSH depletion performance, achieving the cascade amplification of ROS generation.

**Fig. 4:**
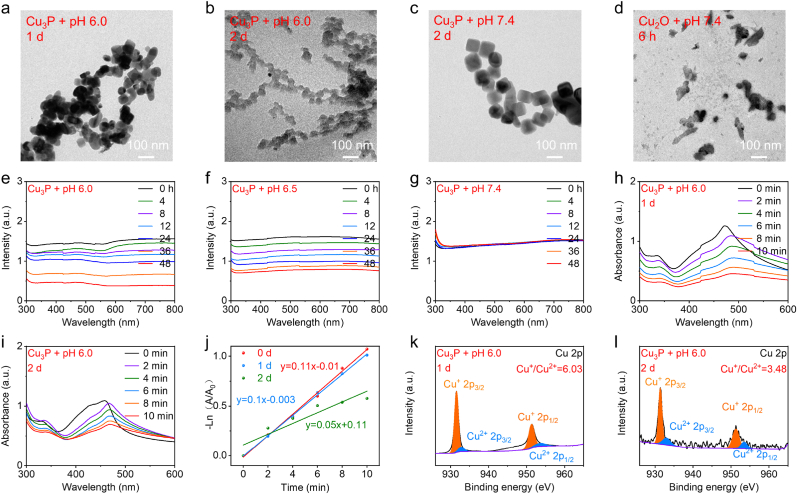
(a–d) Determination of the morphology change of Cu_3_P and Cu_2_O during the degradation process. (e–g) Absorption changes of Cu_3_P and Cu_2_O during the degradation process. (h–j) During the degradation process, the sonodynamic activity of Cu_3_P undergoes a change. (k, l) The degradation mechanism evaluation of Cu_3_P through XPS spectrum.

We further examined the degradation behaviors of Cu_3_P and Cu_2_O. The obvious degradation of Cu_3_P was observed at pH 6.0. As presented in Fig. 4a and b, as the incubation increases, the obvious fractionlet was detected in the TEM image of Cu_3_P after 1 day and 2 days. However, the degradation time of Cu_2_O was faster than that of Cu_3_P, which only need 6 h (Fig. S5). More importantly, the fast degradation of Cu_2_O was also detected at pH 7.4, which revealed the fragmentation of Cu_2_O after incubation of only 6 h (Fig. 4d). This phenomenon indicated that Cu_2_O nanocubes was not suitable for the biomedical applications owing to the fast degradation in normal tissues. In contrast, no significant morphology change of Cu_3_P was detected in the normal physiological conditions (Fig. 4c), which still maintains the cubic structure after incubation of 2 days at pH 7.4.

In addition to TEM image, we also utilized the absorption spectrum to determine the degradation behaviors of Cu_3_P and Cu_2_O. As shown in Fig. 4e and f, the significantly decreased absorption spectrum of Cu_3_P can be detected at pH 6.0 and 6.5 during the degradation process. In the absorption spectrum of Cu_2_O, it was also noted that the absorption intensity decreased with longer incubation times (Fig. S6a). However, the decreased absorption intensity of Cu_2_O was also detected at pH 7.4 (Fig. S6b). The photographs of Cu_2_O solution at pH 6.0 and 7.4 also indicated the degradation of Cu_2_O, which revealed that the solution color of Cu_2_O changed from yellow to colorless and transparent (Fig. S7). For comparison, the colorless and transparent solution of Cu_3_P was also observed at pH 6.0 after degradation (Fig. S8), while no significant color change of Cu_3_P solution can be observed at pH 7.4, illustrating that the degradation of Cu_3_P cannot occur in the normal physiological conditions. Apart from color change of solution, the absorption spectrum of Cu_3_P also demonstrated that the degradation of Cu_3_P cannot occur at pH 7.4 (Fig. 4g). These results forcefully manifested that the phosphating process of Cu_2_O can avoid the degradation of Cu_2_O in the neutral condition.

We then investigated whether the degradation of Cu_3_P would influence the sonodynamic activity of Cu_3_P. The US-activated ROS production through Cu_3_P exhibited no obvious attenuation after 1 day (Fig. 4h and i), manifesting that the degradation of Cu_3_P would not influence the sonodynamic activity of Cu_3_P within 1 day. However, the decreased sonodynamic activity of Cu_3_P was detected after the degradation of 2 days (Fig. 4j), suggesting that the SDT could be performed at the time point of 1 day. The degradation mechanism of Cu_3_P was then investigated through measuring the XPS spectrum. As depicted in Fig. 4k and l, the content of Cu^+^ gradually decreases as the degradation occurs, while the content of Cu^2+^ gradually increases. This indicates that during the degradation process, Cu^+^ is oxidized to Cu^2+^, which subsequently leads to the collapse of the Cu_3_P structure. The structural collapse was also demonstrated by the XRD pattern, which indicated that the diffraction peaks of Cu_3_P become weak as the incubation time prolongs (Fig. S9).

**Fig. 5:**
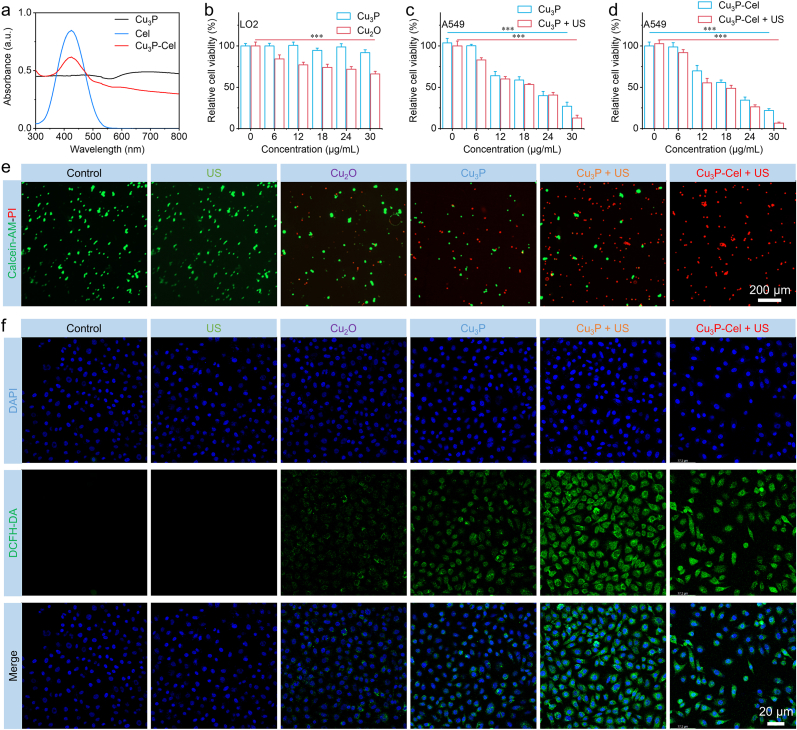
(a) Absorption spectrum of Cu_3_P, Cel, and Cu_3_P-Cel. (b–d) Cytotoxicity of Cu_3_P, Cel, and Cu_3_P-Cel against LO2 or A549 cells with or without US irradiation. (e, f) Live/dead and ROS staining of A549 cells post different treatments. Data are presented as the mean ± SD. (n = 6). ∗∗∗p < 0.001.

Considering that the Cu_3_P possessed the TME-responsive degradation behaviors, we then utilized Cu_3_P to load anticancer drug celastrol (Cel) for the achievement of TME-responsive drug release. The optimal ratio of Cu_3_P to Cel was confirmed by testing the loading rate of Cel under different mass ratios of Cu_3_P to Cel. The results showed that when the mass ratio of Cu_3_P to Cel is 1:2, the loading rate of Cel is the highest, reaching 20.54 % (Fig. S10). The characteristic absorption peak of Cel located at approximately 430 nm (Fig. 5a). After loading, the characteristic absorption peak of Cel was also detected in the absorption spectrum of Cu_3_P-Cel, demonstrating the successful loading of Cel. The loading of Cel will reduce the Zeta potential of Cu_3_P (Fig. S11). The obtained Cu_3_P-Cel also possessed good stability (Fig. S12). The sonodynamic and chemodynamic activities of Cu_3_P would not decrease after the loading of Cel (Figs. S13, S14, S15). In addition, the GSH depletion capability of Cu_3_P was not affected after the loading of Cel (Fig. S16). These results clearly indicated that the loading of Cel would not influence the sonodynamic, chemodynamic, and GSH depletion performances. We then measured the pH-responsive release behavior of Cel. As presented in Fig. S17, no significant drug release can be detected at pH 7.4, suggesting that Cel cannot be released in the normal cells to induce cytotoxicity. In contrast, the significant release of Cel can be observed at pH 6.0 and 6.5, demonstrating the pH-responsive release behaviors. Specifically, the release rate of Cel at pH 6.0 was calculated to be approximately 80 %. A similar phenomenon was observed in the pH-responsive behavior of Cu ions, which indicated that about 60 % can be released at pH 6.0 after 48 h (Fig. S18).

We then investigated the antitumor activity of Cu_3_P-Cel-mediated combination therapy at the cellular level. The significant cytotoxicity of Cu_2_O against normal LO2 cells can be found in Fig. 5b, which indicated that Cu_2_O was not suitable for the biomedical applications. On the other hand, there is no noticeable reduction in the cell viability of LO2 cells when Cu_3_P is incubated, indicating that Cu_3_P is unable to break down in the healthy cells to release Cu ions. The obvious decreased cell viability of A549 cells after being treated with Cu_3_P and Cu_2_O can be detected (Fig. 5c and S19), confirming the excellent antitumor efficiency of Cu_3_P and Cu_2_O. After Cel loading, the cell viability of A549 cells was further decreased, indicating the combination therapy efficiency (Fig. 5d). Almost all A549 cells were dead and the IC_50_ was only 14.9 μg/mL after the treatment of Cu_3_P-Cel + US (Fig. S20). These results clearly illustrated that the SDT/CDT mediated by Cu_3_P-Cel exhibited a higher anticancer efficacy than monotherapy.

We further assessed the anticancer efficacy of Cu_3_P-Cel + US through conducting the staining of live and dead cells. A certain anticancer effectiveness was observed in A549 cells after being treated with Cu_3_P (Fig. 5e). The stronger anticancer efficacy can be found in the Cu_3_P + US group. We then found that the treatment of Cu_3_P-Cel + US can kill all A549 cells (Fig. 5e). The group with Cu_3_P-Cel + US displayed the most intense green fluorescence signal compared to the other groups, indicating that the production of ROS was predominantly due to Cu_3_P-Cel in synergy with SDT and CDT, as illustrated in Fig. 5f. The quantitative analysis of live/dead staining and ROS staining can be found in Fig. S21. It is well known that the damage of mitochondria in cancer cells can be occurred when the ROS was produced, the mitochondria damage level in A549 cells was then evaluated using JC-1 staining. Fig. S22 exhibited that the strongest fluorescence signal can be detected in A549 cells after being treated with Cu_3_P-Cel + US, demonstrating that the strongest ROS production can induce the most severe mitochondria damage. In addition, this phenomenon illustrated that the combined SDT/CDT through Cu_3_P-Cel can trigger an obvious apoptosis of A549 cells. To demonstrate this assumption, we conducted the apoptosis assay of A549 cells after the treatment of Cu_3_P-Cel + US. As depicted in Fig. S23, most apoptotic A549 cells were found in the Cu_3_P-Cel + US group. We also investigated the GSH consumption performance of Cu_3_P at the cellular level. As shown in Fig. S24, the treatment of Cu_3_P-Cel + US can consume a large amount of GSH within tumor cells, demonstrating the GSH consumption ability of Cu_3_P-Cel.

**Fig. 6:**
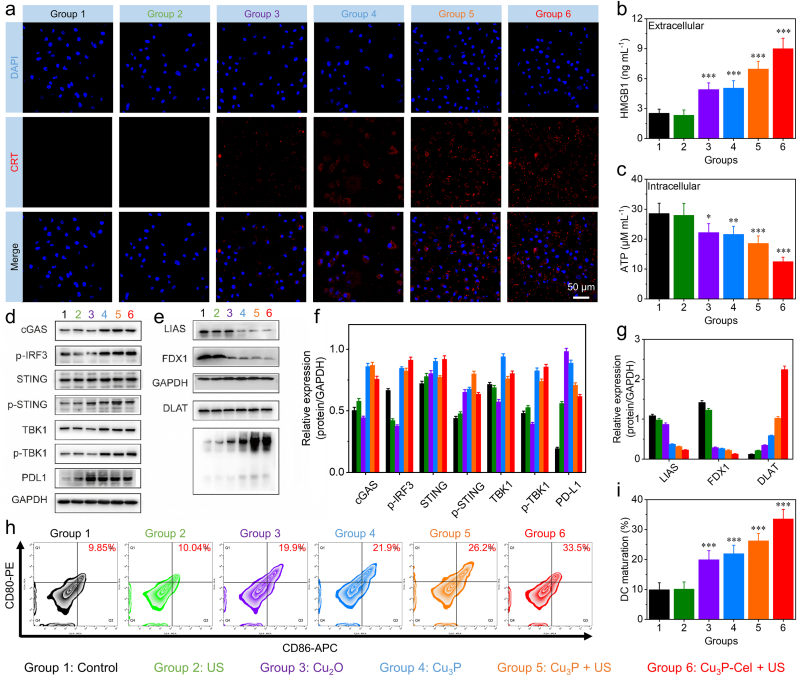
(a) CLSM images of CRT expression in A549 cells after different treatments. (b) Detection of intracellular HMGB1 after different treatments. (c) Detection of extracellular ATP after different treatments. (d, f) A549 cells were treated differently, and the levels of cGAS, IRF3, STING, p-STING, TBK1, p-TBK1, and PD-L1 were analyzed. (e, g) Comparison of LIAS, FDX1, and DLAT in A549 cells through WB analysis post various treatments. (h, i) Measurements of DC maturation level after being treated with Cu_3_P, Cu_3_P + US, and Cu_3_P-Cel + US. Data are presented as the mean ± SD. (n = 3). ∗p < 0.05, ∗∗p < 0.01, and ∗∗∗p < 0.001.

After demonstrating the efficacy of Cu_3_P-Cel-mediated combination therapy in specifically combating tumors, the antitumor mechanism was then investigated through carrying out a series of experiments. With Cu ions present and the TME-responsive degradation behaviors of Cu_3_P in mind, we studied the impact of Cu_3_P on the cuproptosis pathway in A549 cells. In Fig. 6e, the aggregation of DLAT in A549 cells was clearly observed following exposure to Cu_3_P, Cu_3_P + US, and Cu_3_P-Cel + US. In addition to DLAT oligomerization, the decreased expression level of LIAS and FDX1 in A549 cells was also detected after the treatment of Cu_3_P, Cu_3_P + US, and Cu_3_P-Cel + US (Fig. 6g). These three markers forcefully demonstrated that the cuproptosis pathway was activated by Cu_3_P-Cel-mediated combination therapy. To further enhance the credibility of the conclusion, we conducted a cuproptosis inhibition experiment and added a positive control (elesclomol-Cu). As presented in Fig. S25, for the Cu_3_P-Cel group and the elesclomol-Cu group, the expression levels of cuproptosis-related proteins LIAS and FDX1 decreased. Meanwhile, oligomerization of DLAT occurred, confirming the occurrence of cuproptosis. For the Cu_3_P-Cel + TTM group, since Cu ions were chelated by the inhibitor TTM, no significant changes occurred in cuproptosis-related proteins. These results demonstrated that cuproptosis can be induced by Cu_3_P-Cel.

Previous reports indicated that cuproptosis could activate cGAS-STING pathway [52,71]. Based on this situation, we assessed the activation of the cGAS-STING pathway with Cu_3_P-Cel combination therapy. The protein expression levels of STING and TBK1 in the Cu_3_P, Cu_3_P + US, and Cu_3_P-Cel + US groups exhibited no significant change compared to the control group (Fig. 6d). The Cu_3_P, Cu_3_P + US, and Cu_3_P-Cel + US groups exhibited a significant increase in the protein expression levels of p-TBK1, p-STING, cGAS, and p-IRF3 (Fig. 6f), indicating activation of the cGAS-STING pathway by tumor-specific cuproptosis. To further confirm the activation of the cGSA-STING pathway, we further examined the release of IFN-β after different treatments. As presented in Fig. S26, no significant release of IFN-β was detected in the control and US groups, indicating that the cGSA-STING pathway was not activated by the US alone. For comparison, after the treatment of Cu_2_O, Cu_3_P, Cu_3_P + US, or Cu_3_P-Cel + US, the release amount of IFN-β increased significantly, indicating that the cGSA-STING pathway was activated. We then conducted a SITNG inhibition experiment by adding the STING inhibitor H151 to further verify the activation of the STING pathway. As shown in Fig. S27, the expression levels of cGAS, p-STING, and p-TBK1 in Cu_3_P-Cel + H151 group were lower than that in the pristine Cu_3_P-Cel group, demonstrating that the addition of H151 can block the cGAS-STING pathway. This phenomenon also confirmed that Cu_3_P-Cel can activate the cGAS-STING pathway. We also noted that the expression level of PD-L1 was increased in A549 cells after the treatment of Cu_2_O, Cu_3_P, Cu_3_P + US, or Cu_3_P-Cel + US, which could be attributed to the release of Cu ions in the tumor cells. This phenomenon was consistent with the previous reports [72,73], which indicated that the Cu_3_P-Cel-mediated combination therapy could be combined with αPD-L1-induced immune checkpoint blockade to achieve the high-efficiency antitumor immune response.

Many studies have pointed out that the generation of ROS and the induction of cuproptosis may lead to ICD effects [[74], [75], [76], [77]]. The group with Cu_3_P-Cel + US exhibited the most robust CRT signal (Fig. 6a and S28), demonstrating that the most effective therapeutic effects of Cu_3_P-Cel + US are seen with US irradiation. Similar to CRT, Fig. 6b, c showed that the treatment of Cu_3_P-Cel + US led to the highest extracellular HMGB1 level and the lowest intracellular ATP level in A549 cells. It is evident from the findings that the robust ICD effects were triggered by the production of large amounts of ROS and the activation of cuproptosis with Cu_3_P-Cel-triggered combination therapy.

We then explored whether the treatment of Cu_3_P-Cel + US could enhance the maturation level of DCs after demonstrating the successful triggering of ICD and STING pathway. We utilized Cu_3_P-Cel to treat A549 cells, and the US treatment was applied at the same time. After the treatment, the supernatants of A549 cells were collected and added to the DCs. Fig. 6h demonstrated the increase of CD80/CD86 expressions in DCs after being treated with Cu_2_O, Cu_3_P, Cu_3_P + US, or Cu_3_P-Cel + US, manifesting the maturation of DCs can be occurred by integrating ICD/cuproptosis/cGAS-STING-activation. Specifically, Fig. 6i indicated that the Cu_3_P-Cel + US group had the highest percentage of CD80^+^CD86^+^ DCs compared with the other group, which verified that integrating ICD/cuproptosis/cGAS-STING-activation functions in Cu_3_P-Cel could significantly promote DC maturation.

**Fig. 7:**
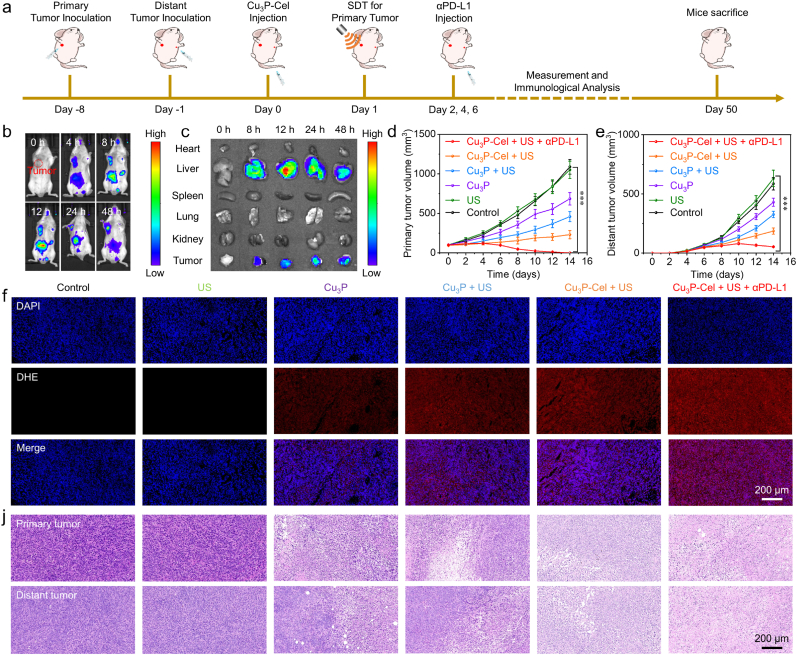
(a) A diagram illustrates the in vitro anticancer treatment steps for Cu_3_P-Cel-mediated combination therapy. (b, c) The accumulation of Cu_3_P-Cel in tumor tissues. (d, e) Mice tumor volume at primary and distant locations post diverse treatments. (f, j) Tumor tissues from different groups were subjected to DHE and H&E staining. Data are presented as the mean ± SD. (n = 5). ∗∗∗p < 0.001.

We then fabricated an animal model simulating the metastasis of cancer (bilateral tumors) and evaluated the anticancer efficacy of Cu_3_P-Cel + US (Fig. 7a). After intravenous injection of Cu_3_P-Cel, the optimal irradiation time point lies in the highest accumulation of nanodrugs in tumors. In determining this time point, we utilized in vivo fluorescence imaging of ICG-labeled Cu_3_P-Cel. Following injection, the fluorescence signal at the tumor site showed a continuous increase, indicating the progressive accumulation of Cu_3_P-Cel in tumors (Fig. 7b). We also found that the most accumulation of Cu_3_P-Cel in tumors was 24 h post-injection (Fig. S29). For a more detailed presentation of the in vivo imaging outcome, ex vivo imaging was conducted. The predominant accumulation of Cu_3_P-Cel in the liver occurred after injecting of 12 h, while the predominant accumulation of Cu_3_P-Cel in the tumors occurred after injecting of 24 h (Fig. 7c and S30). Therefore, we can perform the irradiation of US at the time point of 24 h post-injection. As depicted in Fig. S31, no significant temperature changes can be detected in the tumor tissues during the US irradiation period, indicating that the US irradiation did not produce obvious thermal effects.

We then measured the volume of tumors to assess the anticancer efficacy of Cu_3_P, Cu_3_P-Cel, Cu_3_P + US, Cu_3_P-Cel + US, Cu_3_P-Cel + US + αPD-L1. The Cu_3_P alone group showed a certain therapeutic effect against the primary and distant tumors (Fig. 7d and e), which could be attributed to the chemodynamic activity and cuproptosis effect of Cu_3_P. When US irradiation applied, the therapeutic effect of primary and distant tumors was enhanced. The decreased primary and distant tumor volume also manifested that Cu_3_P-Cel + US can induce antitumor immune response. This phenomenon also illustrated that the loading of chemotherapy drug could further enhance the antitumor effectiveness. Considering that the release of Cu ions could increase the expression of PD-L1, we then combined the αPD-L1-induced immune checkpoint blockade with SDT/CDT/cuproptosis/cGAS-STING-activation through Cu_3_P-Cel + US + αPD-L1. A high-efficiency therapeutic effect was observed with the combination therapy of Cu_3_P-Cel + US + PD-L1. However, the use of αPD-L1 alone has no significant inhibitory effect on the growth of primary and distal tumors in 4T1 tumor-bearing mice (Fig. S32). This may be due to the low expression level of PD-L1 in tumor cells that have not undergone immune regulation, and the limited proportion of PD-L1 immunosuppressive cells (such as TAMs) in the tumor microenvironment. This leads to αPD-L1 being unable to effectively block the “immune checkpoint” signal, making it difficult to relieve the immunosuppressive state of T cells and thus unable to effectively inhibit tumor growth. Furthermore, the outcomes of TUNEL and H&E staining indicated that tumors exhibited almost complete necrosis after being treated with Cu_3_P-Cel + US + PD-L1 (Fig. 7g and S33). Furthermore, mice that received Cu_3_P-Cel + US + αPD-L1 exhibited a prolonged survival period when compared to the control group (Fig. S34).

Following the successful demonstration of Cu_3_P-Cel-mediated combination therapy's outstanding antitumor effectiveness, we examined the antitumor mechanism of Cu_3_P-Cel + US. We demonstrated that combination therapy can produce ROS. As presented in Fig. 7f, the Cu_3_P alone group exhibited an obvious red fluorescence signal. More ROS can be generated by Cu_3_P + US, Cu_3_P-Cel + US, and Cu_3_P-Cel + US + αPD-L1. As illustrated in Fig. S35, compared with the other groups, the highest ATP and HMGB1 levels can be detected in the Cu_3_P-Cel + US + αPD-L1 group, illustrating that the integration of cuproptosis/cGAS-STING-activation/SDT/CDT with immune checkpoint blockade could elicit stronger ICD effects.

**Fig. 8:**
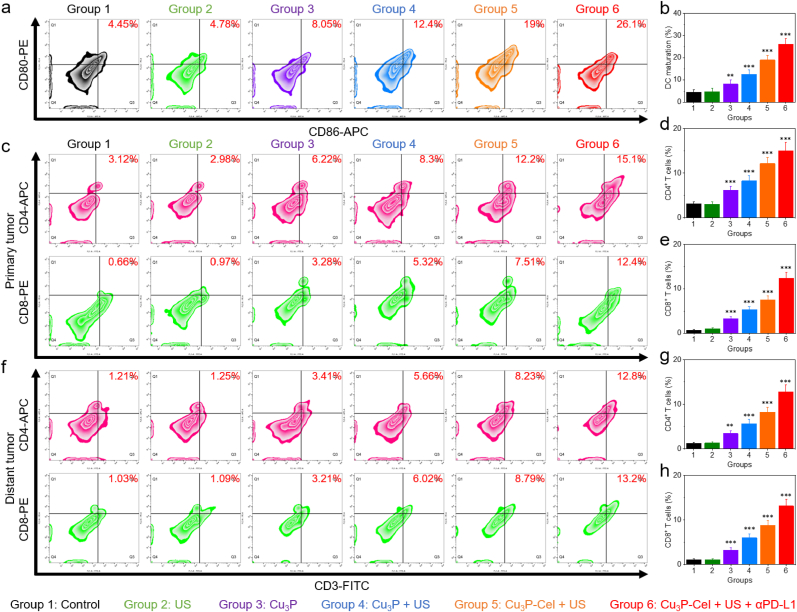
(a–h) Measurements of the level of CD80, CD86, CD4, and CD8 in the lymph nodes and tumors of mice after being treated with Cu_3_P, Cu_3_P + US, Cu_3_P-Cel + US, and Cu_3_P-Cel + US + αPD-L1. Data are presented as the mean ± SD. (n = 3). ∗∗p < 0.01 and ∗∗∗p < 0.001.

We then investigated if the administration of Cu_3_P-Cel + US + αPD-L1 can promote DC maturation. Using flow cytometry, the levels of CD80/CD86 expressions in the lymph nodes can be evaluated after collecting the single-cell suspensions. As expected, the expression level of CD80/CD86 in Cu_3_P-Cel + US group revealed an obvious increase when compared to the Cu_3_P alone group. Moreover, the highest levels of CD80/CD86 expressions can be found in the Cu_3_P-Cel + US + αPD-L1 group (Fig. 8b), manifesting that the integration of cuproptosis/cGAS-STING-activation/SDT/CDT with immune checkpoint blockade could significantly improve antigen presentation. To further confirm the activation of DC maturation, we examined the secretion of immune-related cytokines. As depicted in Fig. S36, compared with the control and US treatment groups, the secretion of immune-related cytokines such as IFN-γ, TNF-α, and IL-6 in the Cu_3_P-Cel + US + αPD-L1 group has significantly increased.

We then assessed the level of CD4/CD8 in tumors to clarify the activation of T cells through integrating cuproptosis/cGAS-STING-activation/SDT/CDT with immune checkpoint blockade through Cu_3_P-Cel + US + PD-L1. As presented in Fig. 8c–e, the primary tumors treated with Cu_3_P-Cel + US + PD-L1 exhibited an enhanced level of CD4/CD8. The similar enhancement effect of CD4/CD8 can also be occurred in distant tumors and spleen after the treatment of Cu_3_P-Cel + US + PD-L1 (Fig. 8f–h and S37). The activation of immunotherapy was found to be amplified when Cu_3_P-Cel + αPD-L1 was combined with US, suggesting a potential synergistic effect. Collectively, the boosting of anticancer immune responses through promoting DC maturation and activating T cell can be achieved by the treatment of Cu_3_P-Cel + US + PD-L1 because the released Cu ions and Cel can lead to amplified ROS yield, cuproptosis, and cGAS-STING activation.

We finally evaluated the biosafety of the treatment of Cu_3_P-Cel + US + PD-L1. This treatment approach cannot induce weight loss of mice (Fig. S38), suggesting that these treatments were safety. We also studied the biodistribution and excretion pathways of Cu_3_P-Cel in the body. Fig. S39a showed that Cu_3_P-Cel mainly accumulates in the liver 24 h after injection, due to the capture of the reticuloendothelial system. Importantly, no obvious Cu signal was observed in these organs after 14 days, indicating that Cu_3_P-Cel was almost completely cleared. As presented in Fig. S39b, the Cu content in both feces and urine gradually declined to nearly undetectable levels after 14 days, suggesting that Cu_3_P-Cel were predominantly eliminated from the mice via hepatic and renal excretion pathways. Subsequently, a series of blood examination was carried out after collecting the blood of mice. All blood markers were within the normal range, as shown in Fig. S40. Once the 14-day treatment regimen was completed, the mice were euthanized and their major organs were collected. Then, the organs were H&E stained to validate the biocompatibility of Cu_3_P-Cel + US + αPD-L1 application. As illustrated in Fig. S41, the good biosafety and histocompatibility of the treatment of Cu_3_P-Cel + US + PD-L1 can be demonstrated by the negligible abnormalities of the major organs.

## 3 Conclusions

In summary, we report the integration of ROS-mediated-ICD/cuproptosis/cGAS-STING-activation with ICB to realize the cascade amplification of antitumor immune response. To demonstrate this concept, we synthesized biodegradable Cu_3_P nanocubes from Cu_2_O nanocubes. Because Cu_3_P nanocubes exhibit TME-responsive degradation behaviors, Cel, a commonly used clinical drug, was attached to the nanocubes for targeted drug release in tumors. The sonodynamic activity of the Cu_3_P nanocubes was higher than that of the Cu_2_O nanocubes because of the decreased bandgap. Reversing the immunosuppressive TME is possible through the cascade amplification of ROS generation efficiency and tumor-specific cuproptosis. Cuproptosis specific to tumors can induce ICD and increase the expression of PD-L1 in tumor cells, enhancing the effectiveness of ICB-mediated tumor therapy. Moreover, Cu_3_P-facilitated cuproptosis initiated the cGAS-STING pathway and enabled the cascade amplification of antitumor immune response.

## 4 Experimental section

### 4.1 Preparation of Cu_2_O

Sodium dodecyl sulfate (SDS) (0.26 g) and CuSO_4_ (1.2 M) were mixed and stirred for 10 min. Following that, NaOH solution (4.8 M) was included, and the stirring was carried out for 5 min. Subsequently, 1 mL of ascorbic acid (SA) (1.1 M) was included and stirring was carried out for 5 min. Following a 24-h period, the precipitation was gathered through centrifugation at 12000*g* for 5 min and subsequently rinsed with DI water thrice to acquire Cu_2_O.

### 4.2 Preparation of Cu_3_P

0.3 g Cu_2_O and 1.5 g sodium phosphate were fully mixed in an agate mortar, and the mixed powder was annealed at 200 or 300 °C for 3 h under flowing argon protection. The collected bulk products were fully ground with a grinding rod, then washed alternately with DI water and ethanol.

### 4.3 Preparation of Cu_3_P-Cel

Cel was dissolved in ethanol. The Cu_3_P NPs solution was magnetically stirred with the Cel solution (Cu_3_P: Cel = 1 : 2 (mass ratio)) overnight. The precipitation was collected by centrifugation, and the excess Cel was removed by washing with ethanol several times to obtain Cu_3_P-Cel.

### 4.4 Sonodynamic, chemodynamic, and GSH depletion performances

For sonodynamic measurements, DPBF at a mass of 20 μg was added to Cu_2_O, Cu_3_P and Cu_3_P-Cel solution (0.3 mg/mL). US irradiation (50 kHz, 1.0 W/cm^2^, 50 % duty cycle) was performed. In addition, we also utilized ESR spectrum to detect generated ^1^O_2_ in the presence of Cu_2_O, Cu_3_P and Cu_3_P-Cel using TEMP as the trapping agent. For chemodynamic measurements, TMB (10 μL) was added to Cu_2_O, Cu_3_P and Cu_3_P-Cel solution (0.3 mg/mL). The absorption spectrum was used to measure the absorbance of TMB. For GSH consumption measurements, the absorption curves of the mixture were measured after mixing Cu_3_P or Cu_3_P-Cel, GSH, PBS, and DTNB.

### 4.5 MTT assay, ROS detection, JC-1 staining, and live/dead staining

We purchased LO2 and A549 cells from Cell Bank, Chinese Academy of Sciences. To assess the cytotoxicity of Cu_2_O, Cu_3_P and Cu_3_P-Cel, cells were seeded and exposed to these treatments in the presence of US irradiation (50 kHz, 1.0 W/cm^2^, 50 % duty cycle). Finally, the cytotoxicity was evaluated by a standard MTT assay according to the previous report [78]. A549 cells were initially seeded in the confocal plates (2 × 10^5^ cells) or 6-well plates (1 × 10^5^ cells) and treated with PBS, US, Cu_2_O, Cu_3_P, Cu_3_P + US, or Cu_3_P-Cel + US. After different treatments, cells were collected for in vitro ROS detection, JC-1 staining, and live/dead staining. For ROS detection, DCFH-DA and DAPI were utilized as probe to stain ROS and cell nucleus, respectively. For live/dead staining, PI and Calcein-AM were utilized as probe to stain dead and live cells, respectively.

### 4.6 In vitro detection of ICD biomarkers

A549 cells were initially seeded in the 12-well plates (5 × 10^4^ cells) and treated with PBS, US, Cu_2_O, Cu_3_P, Cu_3_P + US, or Cu_3_P-Cel + US. Based on the provided protocols, after different treatments, we utilized confocal microscope and flow cytometry to detect CRT exposure level. In addition, ATP determination kit and HMGB1 ELISA kit were utilized to detect the ATP and HMGB1 levels in A549 cells after different treatments.

### 4.7 Western blotting

After seeding A549 cells in the 6-well plates, we added the PBS, Cu_2_O, or Cu_3_P into the cells. PBS, Cu_2_O, or Cu_3_P with US irradiation were also performed for WB analysis. The cell lysates incubated with antibodies to LIAS (1:2000, proteintech), FDX1 (1:1000, proteintech), DLAT (1:2000, proteintech), TBK1 (1:2000, ABclonal), STING (1:2000, ABclonal), IRF3 (1:1000, ABclonal), Phospho-TBK1 (1:500, ABclonal), Phospho-STING (1:1000, ABclonal), cGAS (1:1000, ABclonal), PD-L1(1:1000, proteintech), or GAPDH (1:50000, ABclonal) were analyzed and detected by a chemiluminescent imaging system.

### 4.8 In vitro evaluation of DC maturation

A549 cells were seeded in 6-well plates and then exposed to different treatments, including control, US, Cu_2_O, Cu_3_P, Cu_2_O + US, and Cu_3_P + US. BMDCs were obtained from bone marrow-derived cells treated with GM-CSF for one week. The supernatants of 4T1 cells in each group were collected and added to BMDCs. We then utilized CD80-PE, CD86-APC, and CD11b-FITC to stain BMDCs, which were then measured by a flow cytometry.

### 4.9 In vivo antitumor effect

All animal experiments were carried out under the permission by Institutional Animal Care and Use Committee of Shanghai University (SYXK 2019-0020). At day −8, 10^6^ 4T1 cells were subcutaneously injected into the left axilla of mice to construct the primary tumors. Then, at day −1, 10^6^ 4T1 cells were subcutaneously injected into the right axilla of mice to construct the distant tumors. Six groups including BS, US, Cu_3_P, Cu_3_P + US, or Cu_3_P-Cel + US, Cu_3_P-Cel + US + αPD-L1 were divided. Cu_3_P or Cu_3_P-Cel was intravenously injected into mice. We collected tumors or lymph nodes for ROS detection or DC maturation detection, respectively. We also collected tumors and spleen for T cell activation detection.

### 4.10 Statistical analysis

All experiments are performed with at least three independent replicates. Data are presented as the mean ± standard deviation (SD). Statistical significance between the experimental group and the control group is calculated with a two-tailed Student's t-test. ∗ denotes a statistical significance (∗p < 0.05, ∗∗p < 0.01, ∗∗∗p < 0.001) between the data of the experimental group and the control group.

## CRediT authorship contribution statement

**Yue Wu:** Methodology, Investigation. **Shangwei Xu:** Methodology, Investigation. **Jinming Cai:** Software, Methodology. **Jinyan Hu:** Investigation, Funding acquisition. **Dengyu Pan:** Project administration, Funding acquisition. **Bijiang Geng:** Writing – review & editing, Writing – original draft, Supervision, Funding acquisition. **Wen Gao:** Writing – original draft, Supervision. **Yun Wu:** Writing – review & editing, Writing – original draft, Supervision.

## Declaration of competing yest

The authors declare no competing interests.

## Declaration of competing interest

The authors declare that they have no known competing financial interests or personal relationships that could have appeared to influence the work reported in this paper.

## Data Availability

Data will be made available on request.
