# Supplementary material for: Biodegradable Cu-based sonozymes for tumor-specific cuproptosis-enhanced sono-immunotherapy through activating cGAS-STING pathway and sensitizing immune checkpoint blockade
Source: Mater Today Bio. 2025 Oct 10;35:102397. doi: 10.1016/j.mtbio.2025.102397 (PMC12550172; doi:10.1016/j.mtbio.2025.102397)
Supplement: Multimedia component 1 [file mmc1.docx]

Supporting Information

**Biodegradable Cu-based sonozymes for tumor-specific cuproptosis-enhanced sono-immunotherapy through activating cGAS-STING pathway and sensitizing immune checkpoint blockade**

Yue Wu^a,b,1^, Shangwei Xu^a,b,1^, Jinming Cai^c,1^, Jinyan Hu^c^, Dengyu Pan^c^, Bijiang Geng^c,^*, Wen Gao^a,b,^*, Yun Wu^a,b,^*

^a^ Department of Thoracic Surgery, Huadong Hospital, Fudan University, Shanghai 200040, China

^b^ Shanghai Key Laboratory of Clinical Geriatric Medicine, Huadong Hospital, Fudan University, Shanghai 200040, China.

^c^ School of Environmental and Chemical Engineering, Shanghai University, Shanghai, 200444, China

*E-mail addresses*: bjgeng1992@shu.edu.cn (B. Geng), gaowen1232021@163.com (W. Gao), a.ore@live.cn (Y. Wu)

^1^ These authors contributed equally to this work.

*Keywords*: Cu_3_P, cuproptosis, cGAS-STING, sonodynamic therapy, immunotherapy


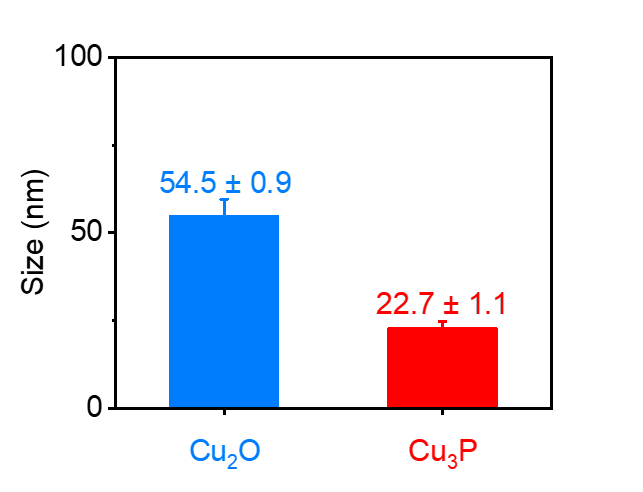


**Fig. S1.** The particle size of Cu_2_O and Cu_3_P. Data are presented as the mean ± SD. (n = 3).


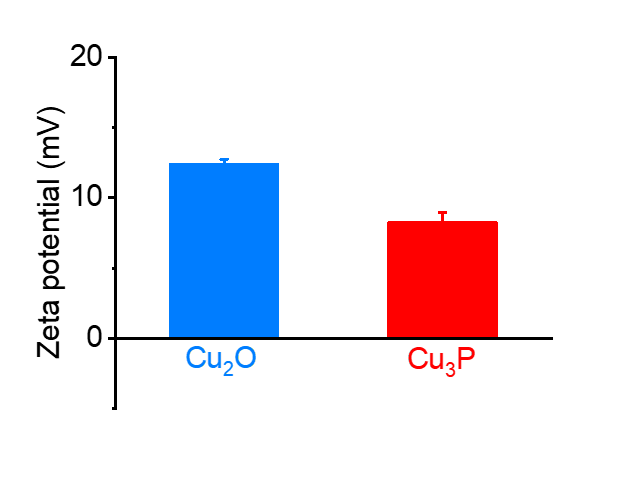


**Fig. S2.** The Zeta potential of Cu_2_O and Cu_3_P. Data are presented as the mean ± SD. (n = 3).


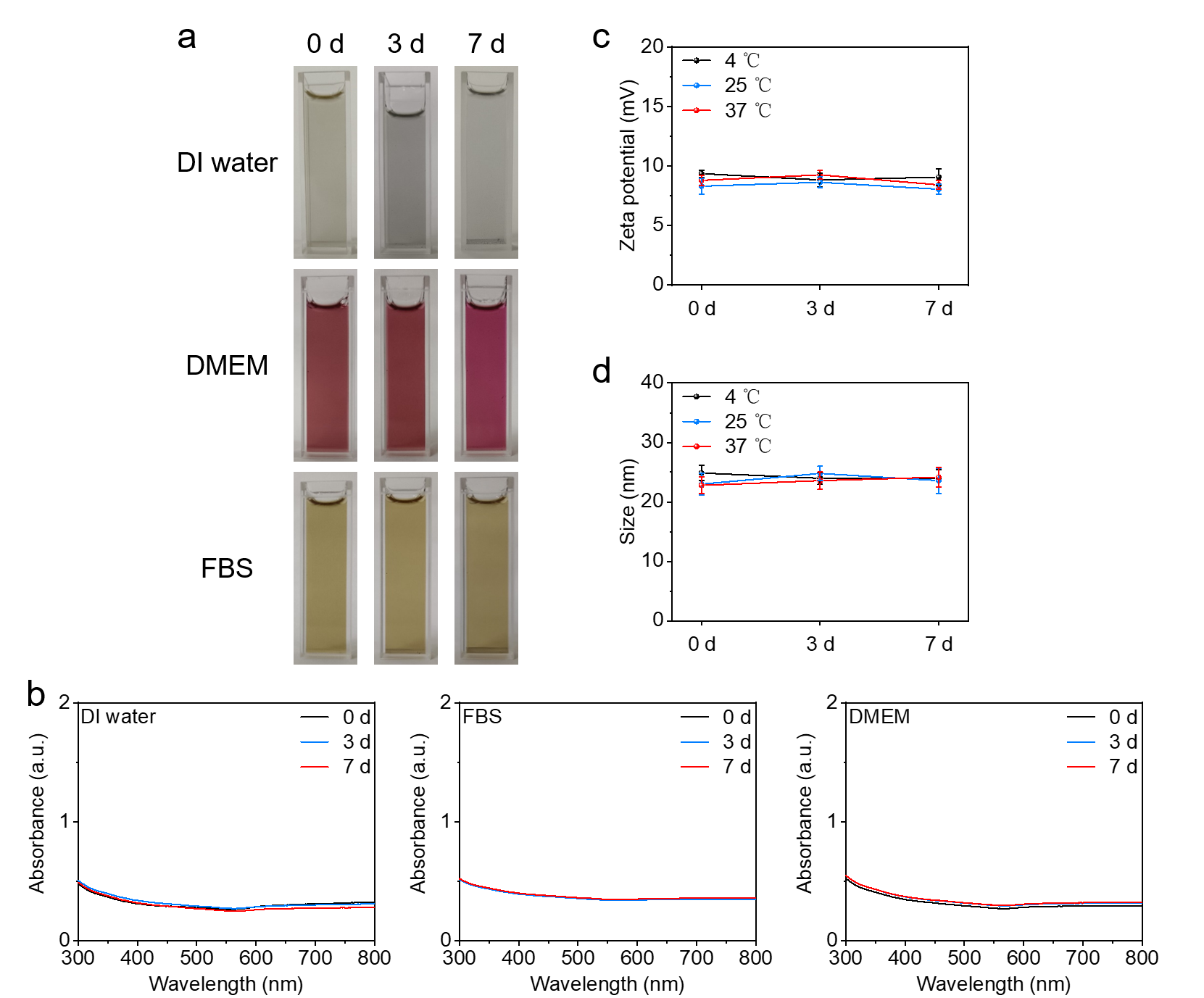


**Fig. S3.** (a, b) Photographs and UV-vis absorption of Cu_3_P dispersed in DI water, FBS, or DMEM for different times. (c, d) Zeta potential and hydrodynamic diameter of Cu_3_P dispersed in 4 ℃, 25 ℃, and 37 ℃ DI water for different times.


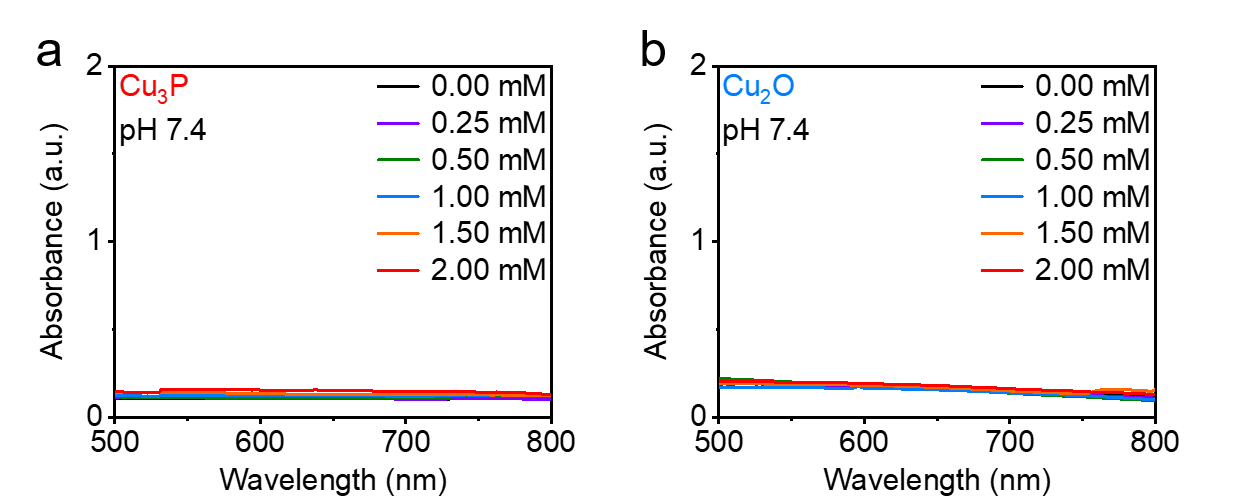


**Fig. S4.** Chemodynamic performance measurements of Cu_3_P (a) and Cu_2_O (b) at pH 7.4.


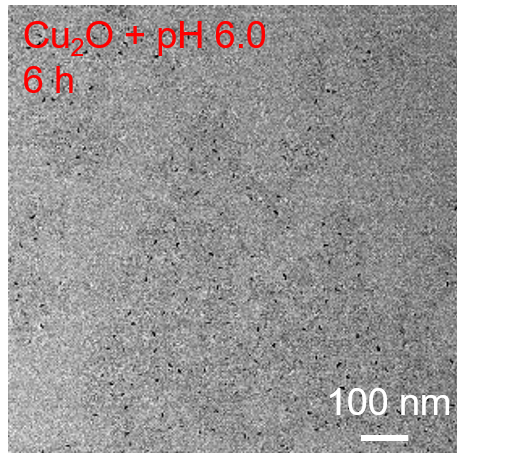


**Fig. S5.** TEM images of Cu_2_O storing at pH 6.0 for 6 h.


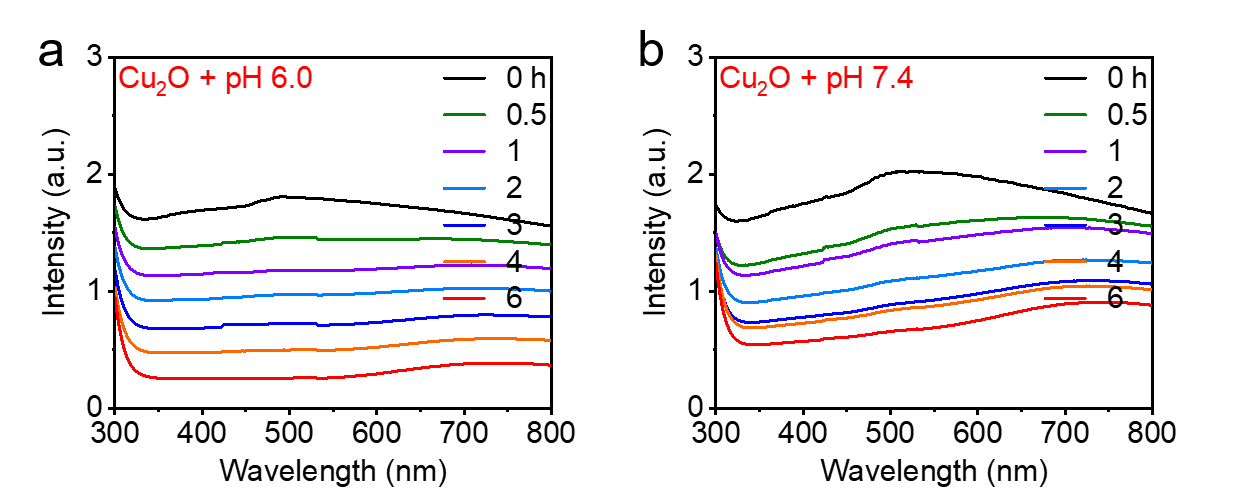


**Fig. S6.** Absorption of Cu_2_O storing at pH 6.0 (a) and 7.4 (b) for different times.


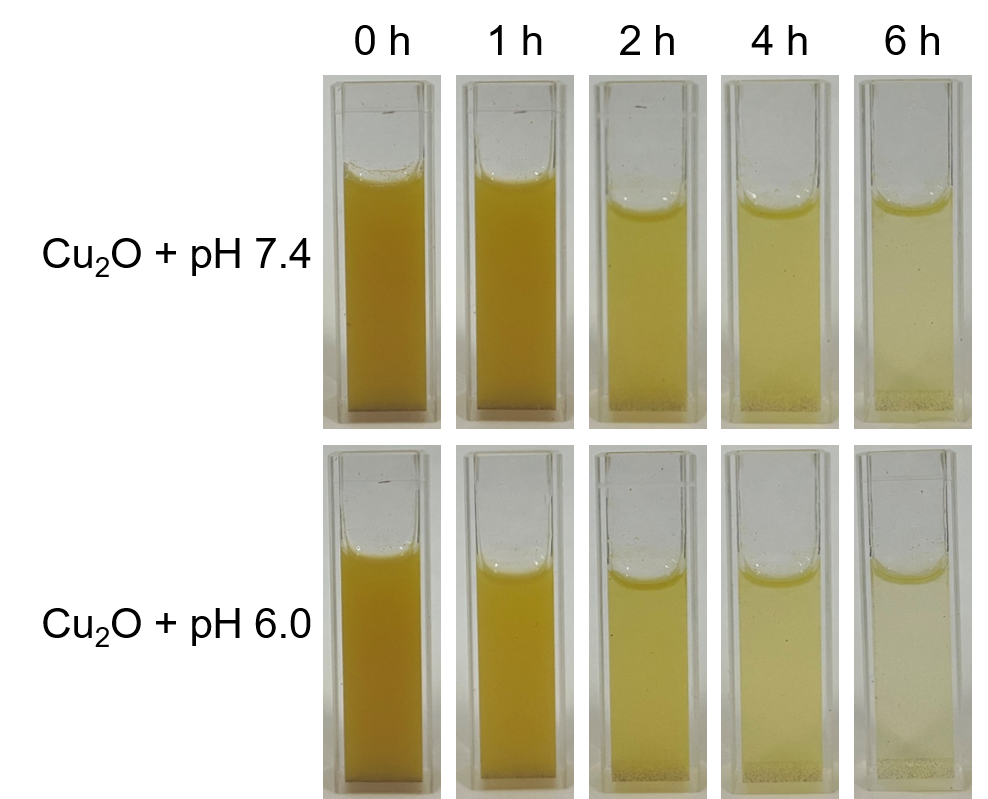


**Fig. S7.** Photographs of Cu_2_O storing at pH 6.0 or 7.4 for different times.


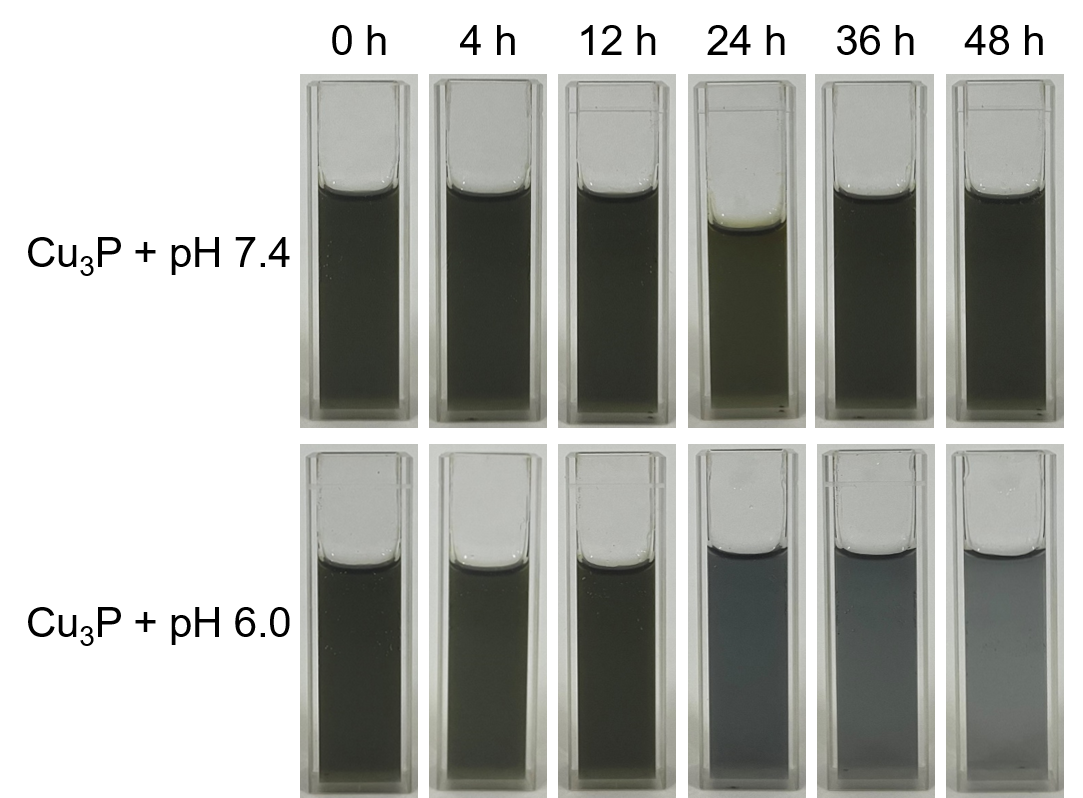


**Fig. S8.** Photographs of Cu_3_P storing at pH 6.0 or 7.4 for different times.


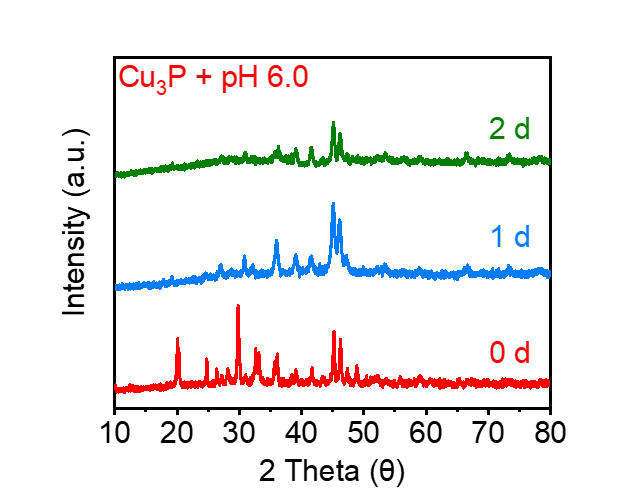


**Fig. S9.** XRD patterns of Cu_3_P storing at pH 6.0 for different times.


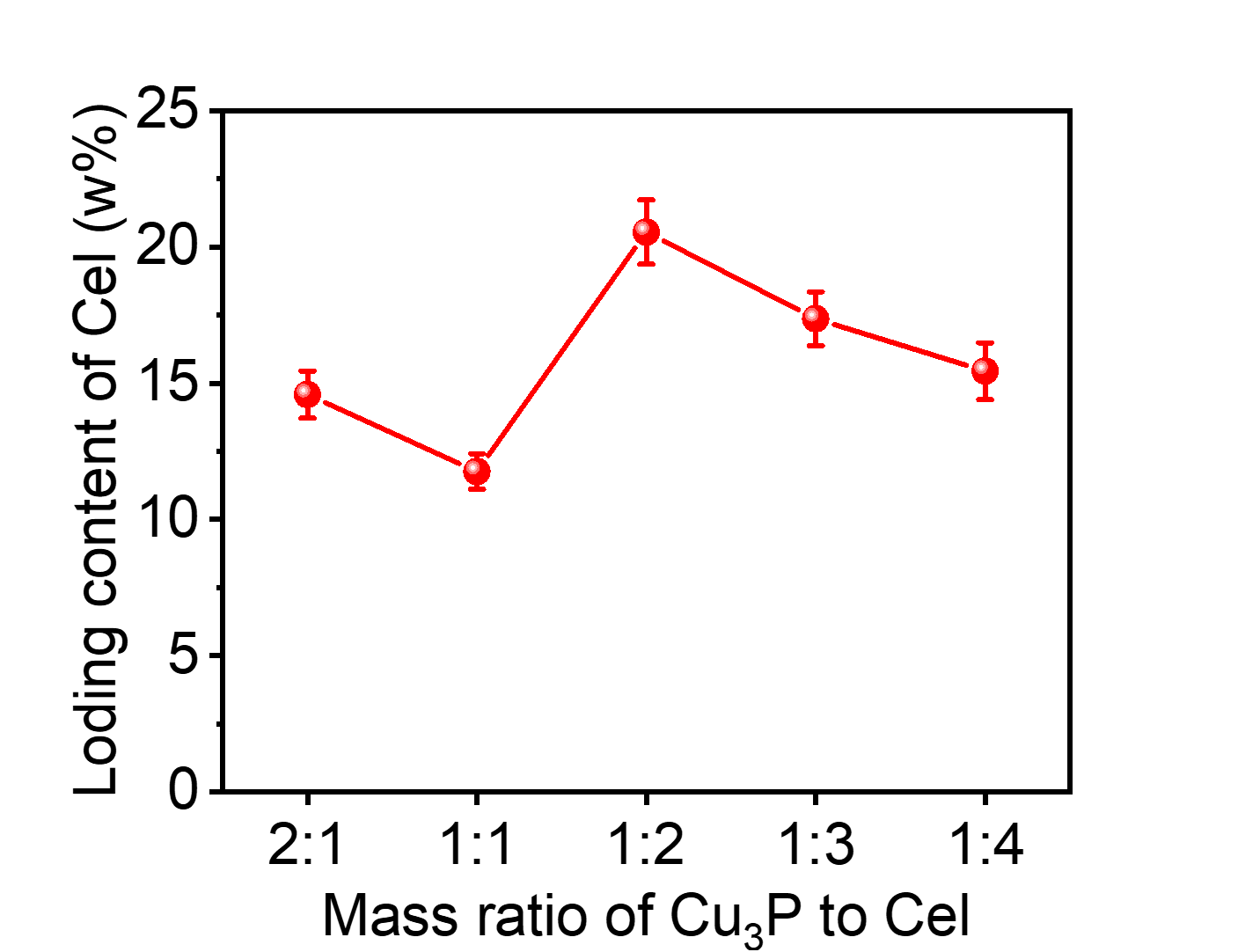


**Fig. S10.** The loading rate of Cel under different mass ratios of Cu_3_P to Cel. Data are presented as the mean ± SD. (n = 3).


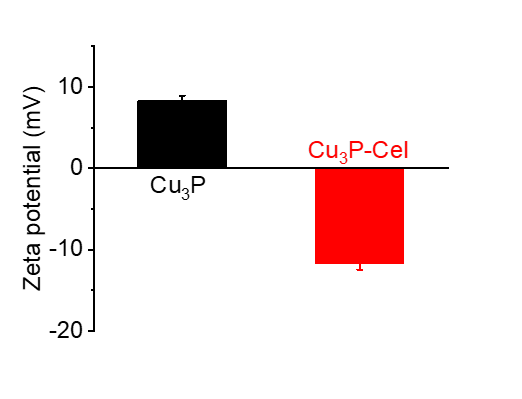


**Fig. S11.** The Zeta potential of Cu_3_P and Cu_3_P-Cel. Data are presented as the mean ± SD. (n = 3).


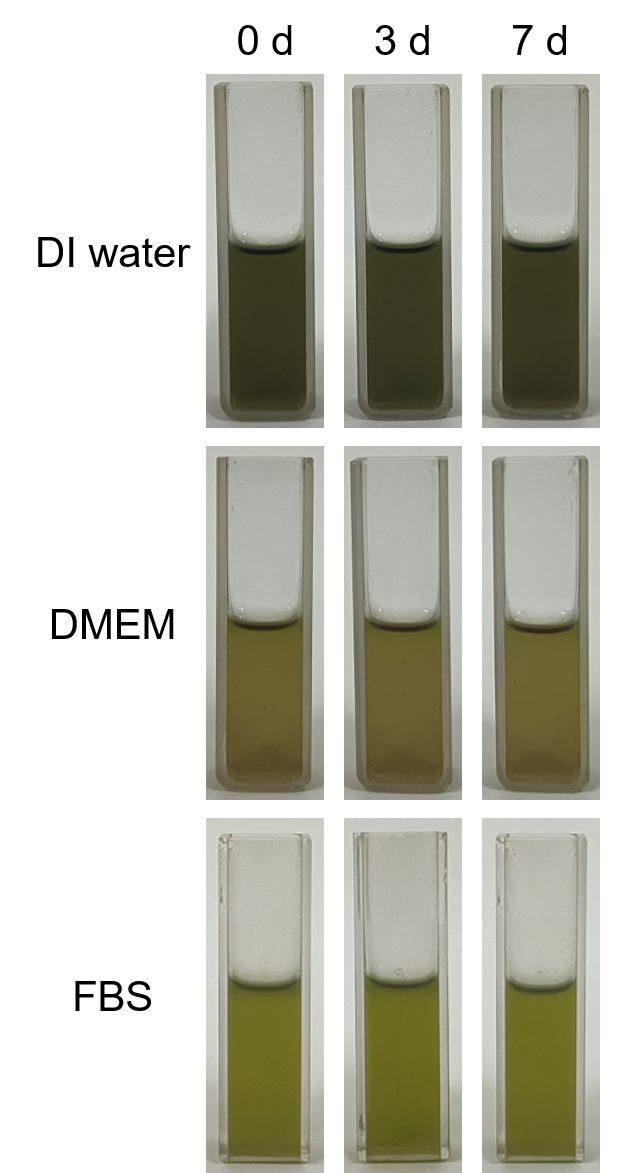


**Fig. S12.** Photographs of Cu_3_P-Cel dispersed in DI water, DMEM, or FBS for different times.


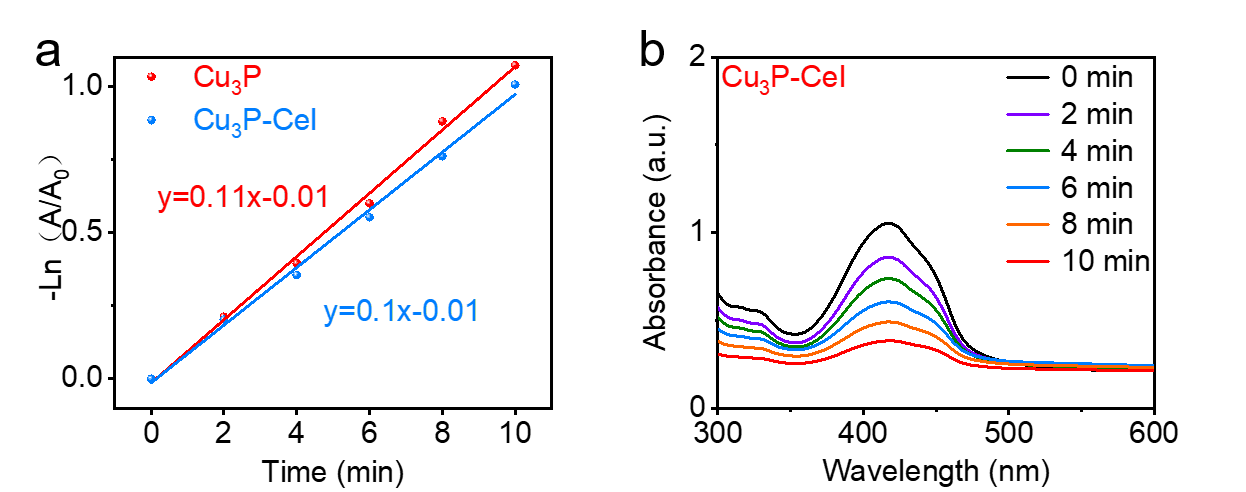


**Fig. S13.** (a) The rate constant of US triggered ^1^O_2_ generation in the presence of Cu_3_P and Cu_3_P-Cel. (b) Time-dependent ^1^O_2_ generation of Cu_3_P-Cel under US irradiation.


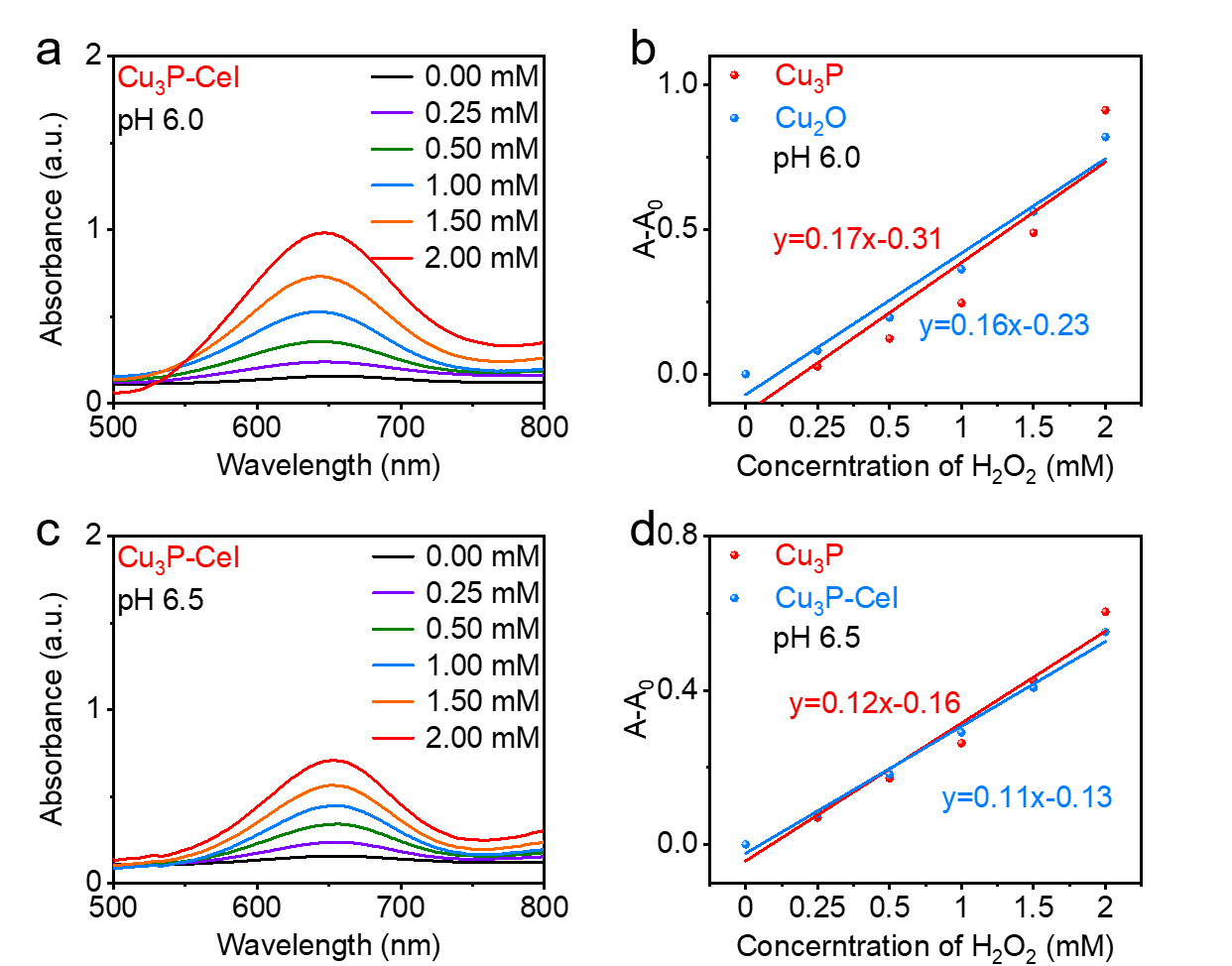


**Fig. S14.** (a) Chemodynamic performance measurements of Cu_3_P-Cel at pH 6.0. (b) Comparison of the chemodynamic effects of Cu_3_P and Cu_3_P-Cel at pH 6.0. (c) Chemodynamic performance measurements of Cu_3_P-Cel at pH 6.5. (d) Comparison of the chemodynamic effects of Cu_3_P and Cu_3_P-Cel at pH 6.5.


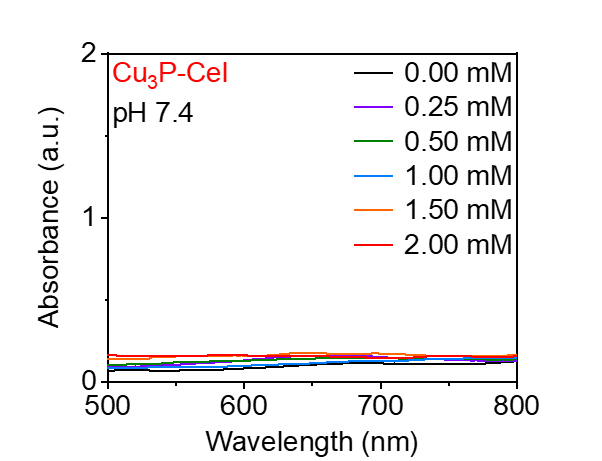


**Fig. S15.** Chemodynamic performance measurements of Cu_3_P-Cel at pH 7.4.


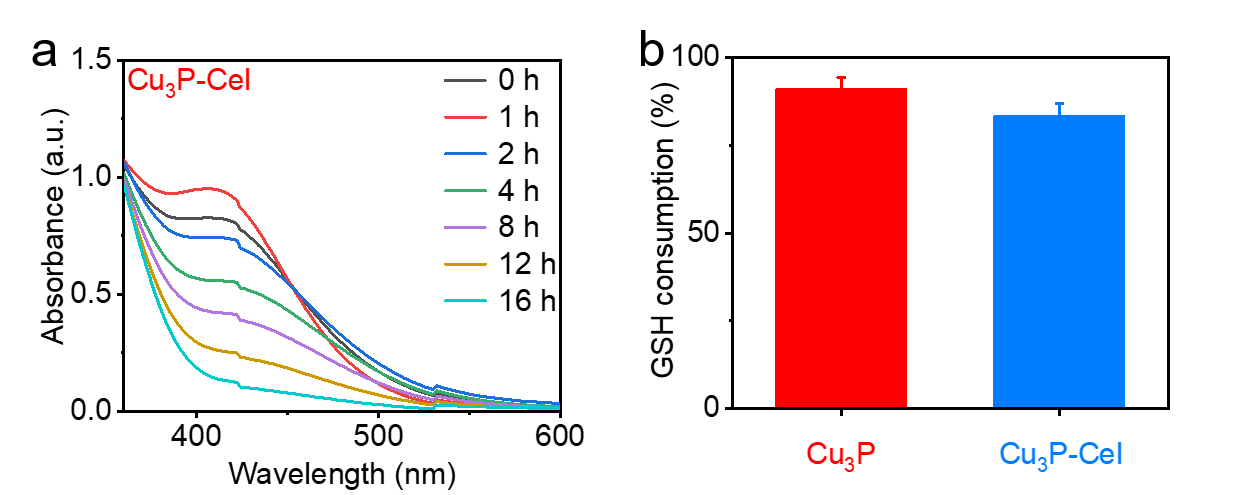


**Fig. S16.** GSH depletion activity evaluation of Cu_3_P-Cel. Data are presented as the mean ± SD. (n = 3).


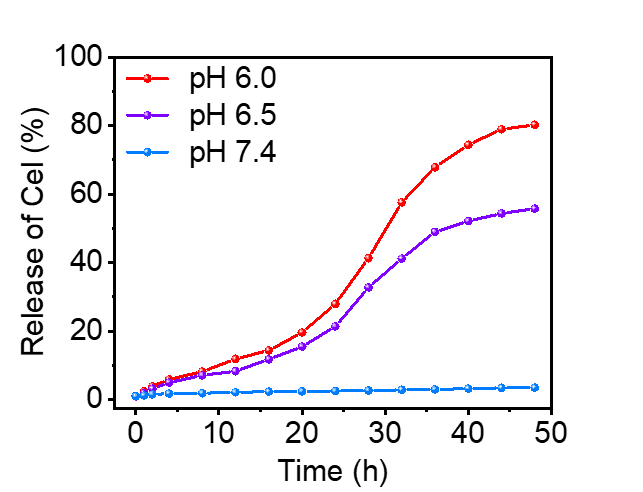


**Fig. S17.** Cel release rate of Cu_3_P-Cel in different pH at different times.


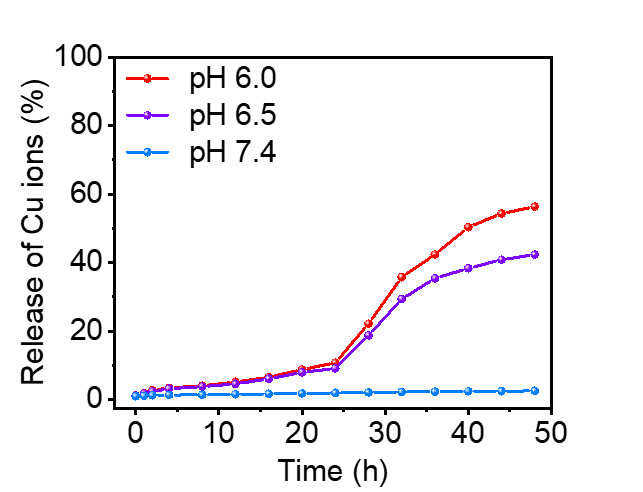


**Fig. S18.** Cu ion release rate of Cu_3_P-Cel in different pH at different times.


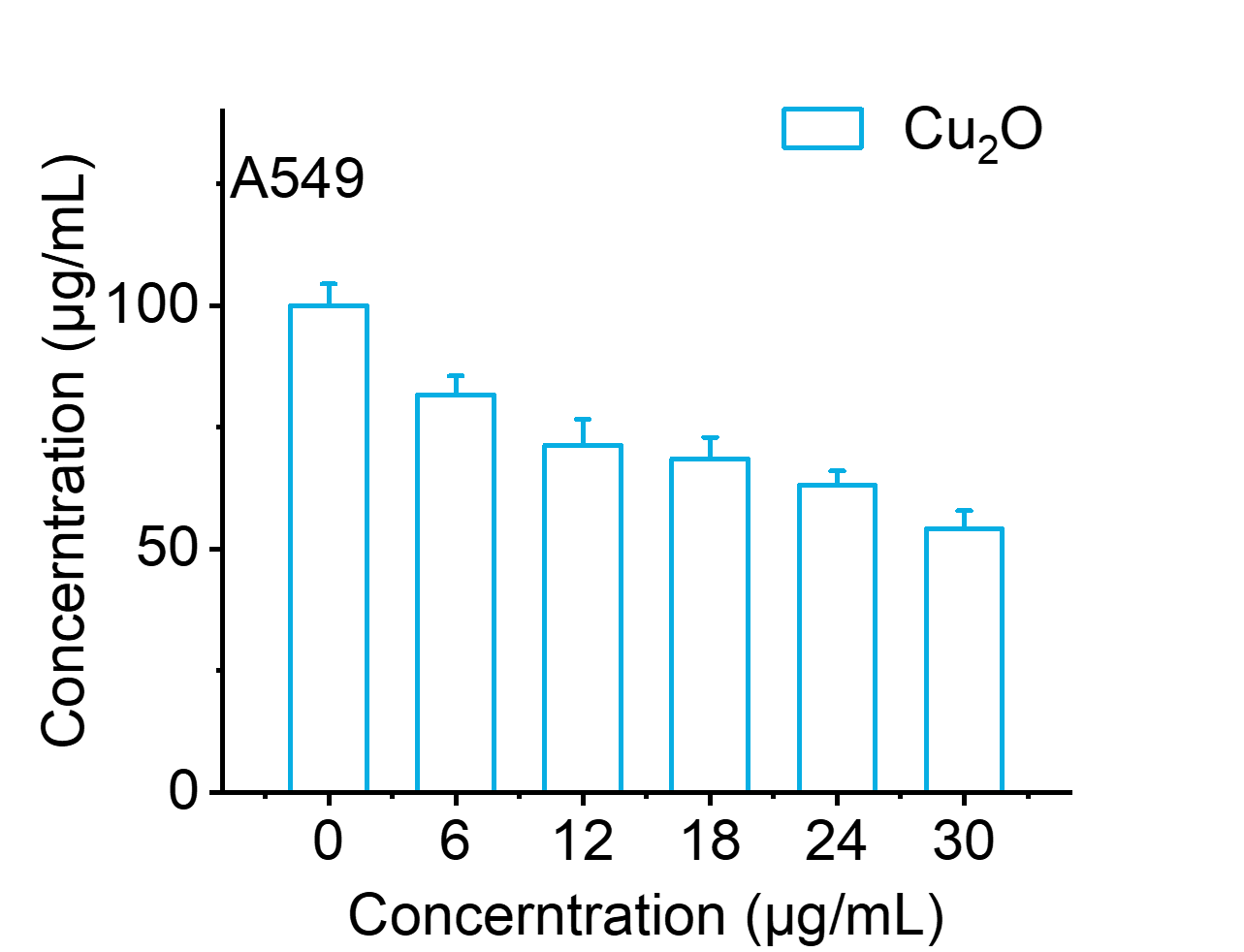


**Fig. S19.** Cell viability of A549 cells treated with Cu_2_O at varied concentrations. Data are presented as the mean ± SD. (n = 6).


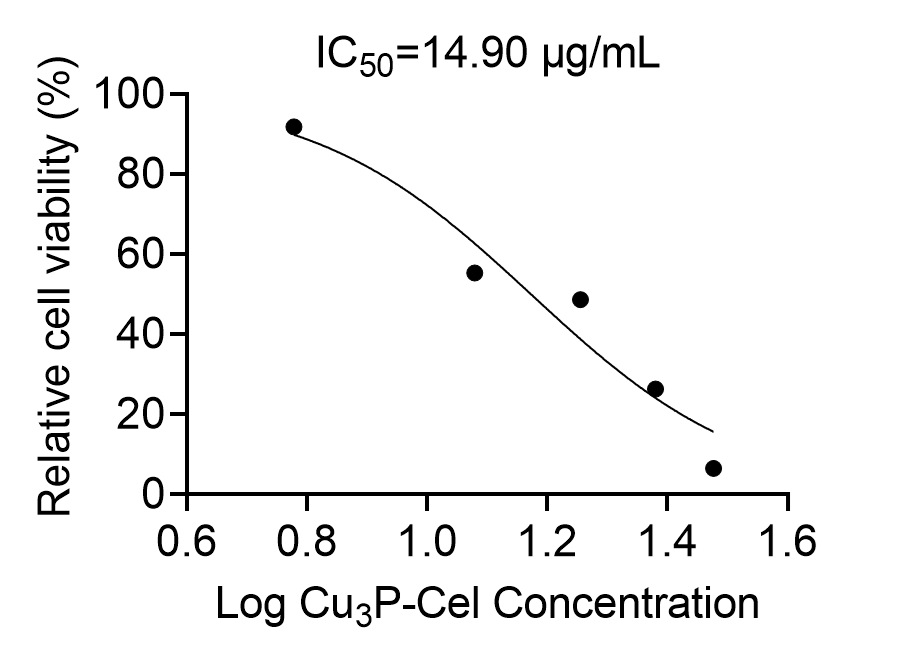


**Fig. S20.** The inhibitory concentration curve of A549 tumor cells fitted by GraphPad Prism after treatment with Cu_3_P-Cel + US.


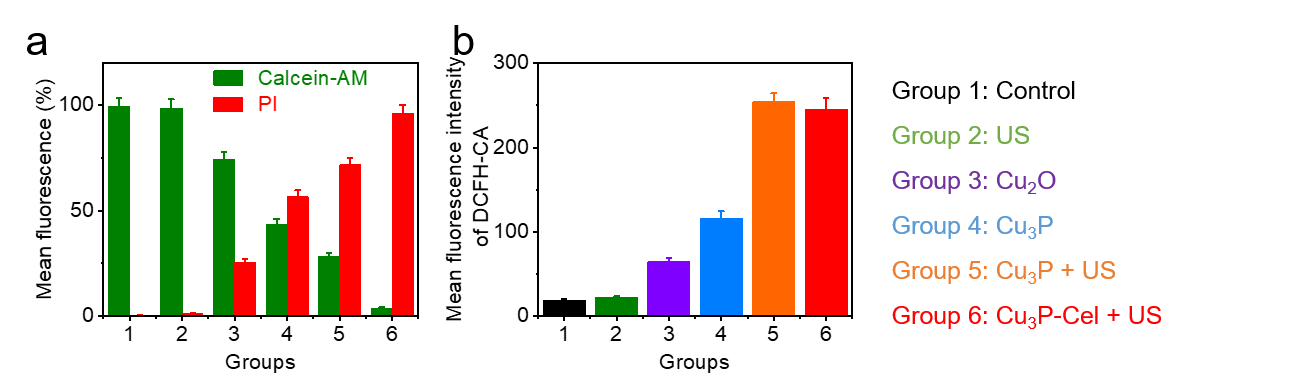


**Fig. S21.** (a) Fluorescence quantitative analysis of live/dead staining. (b) Fluorescence quantitative analysis of ROS staining. Data are presented as the mean ± SD. (n = 3).


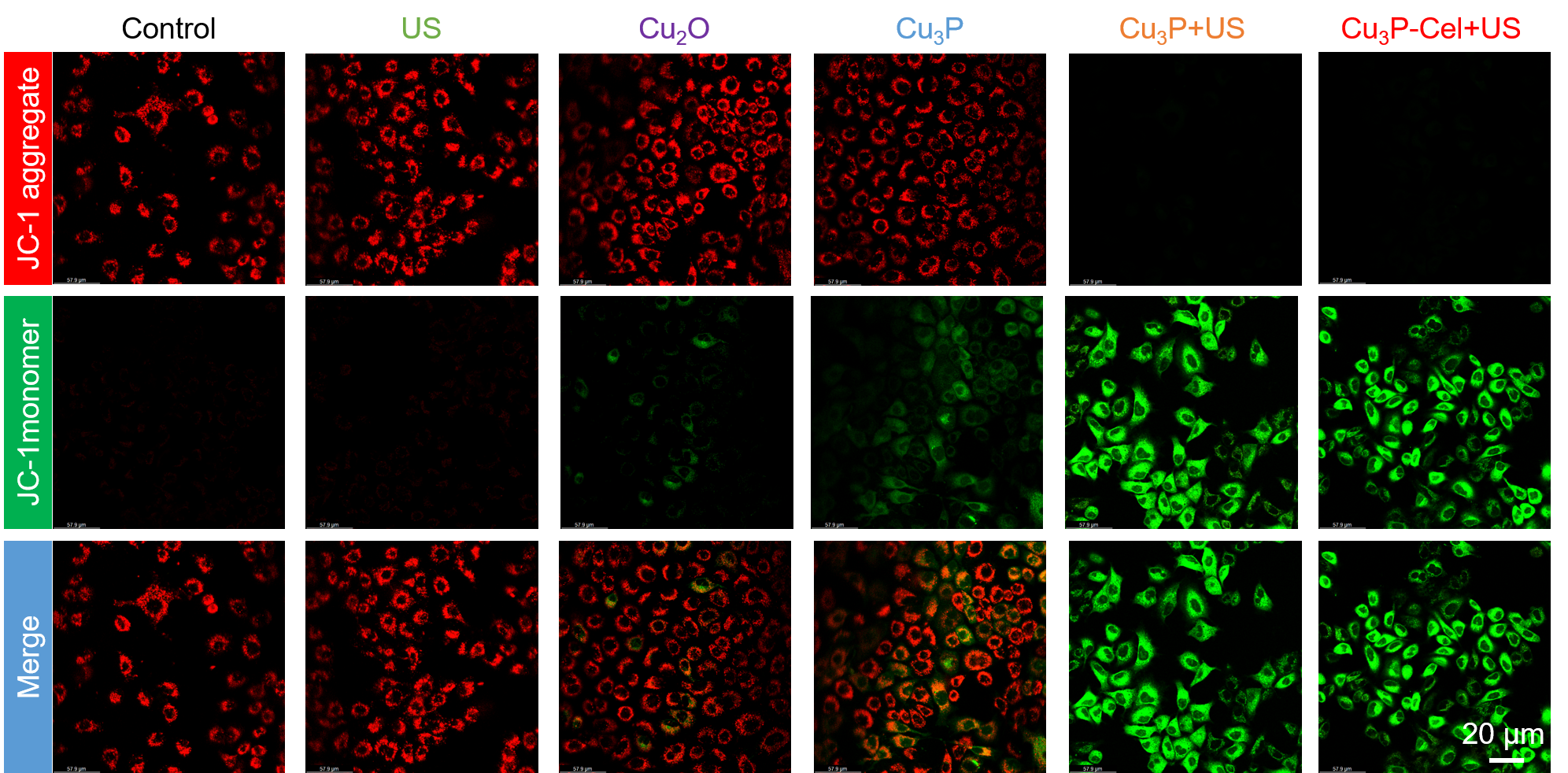


**Fig. S22.** JC-1 staining of A549 cells after different treatments.


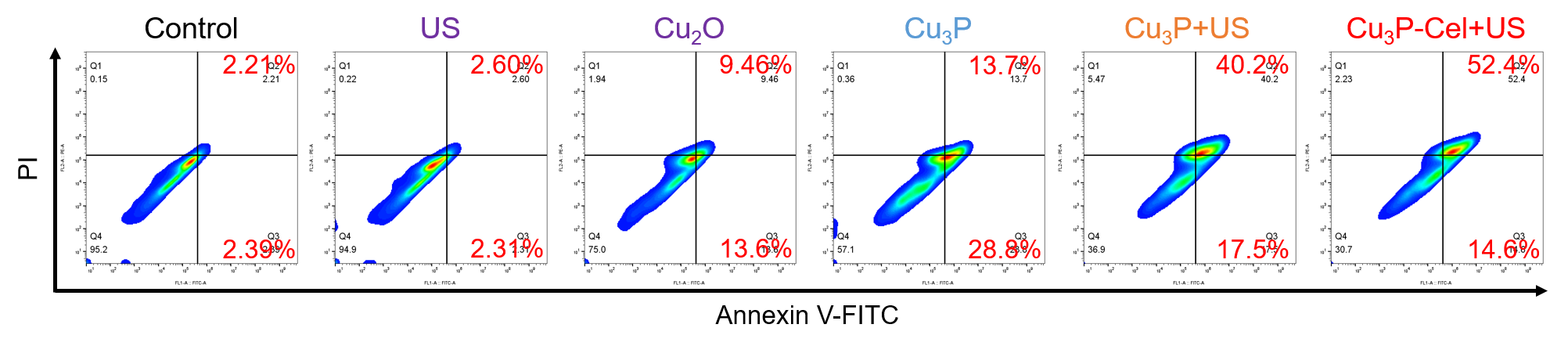


**Fig. S23.** Flow cytometry apoptosis assay of A549 cells after different treatments.


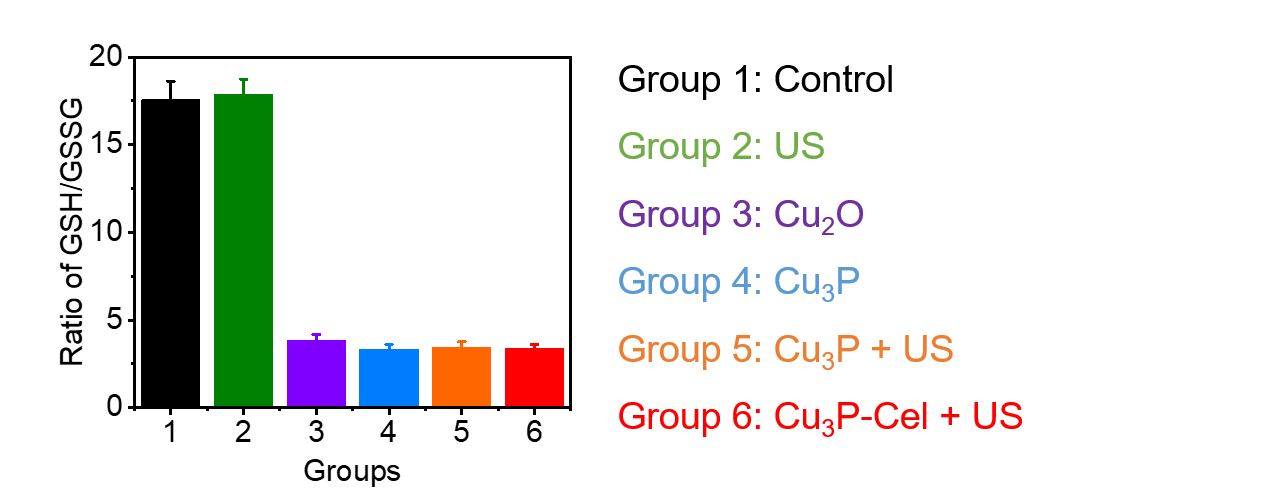


**Fig. S24.** Ratio of GSH to GSSG in A549 cells after different treatments.


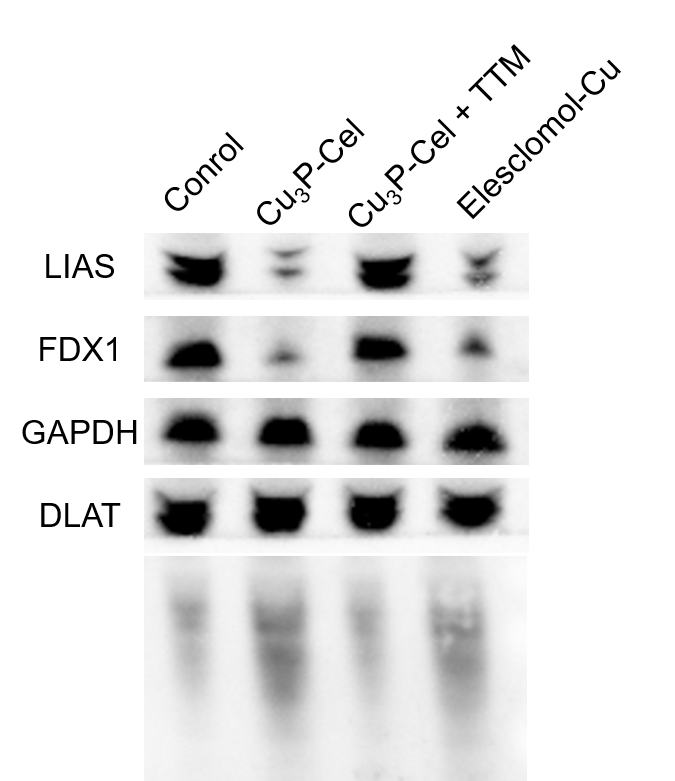


**Fig. S25.** WB analysis of LIAS, FDX1, and DLAT oligomerization in A549 cells after different treatments.


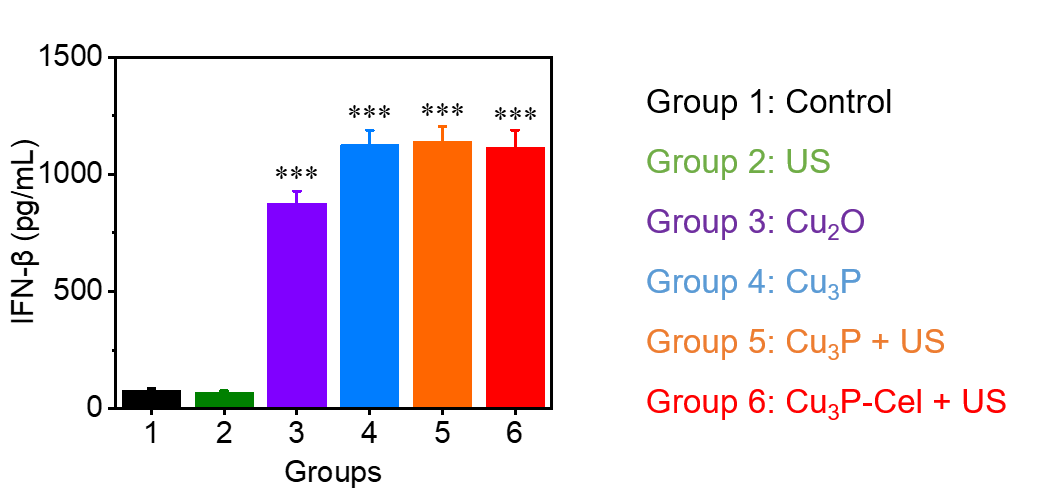


**Fig. S26.** Measurements of IFN-β level after different treatments. Data are presented as the mean ± SD. (n = 3). ***p < 0.001.


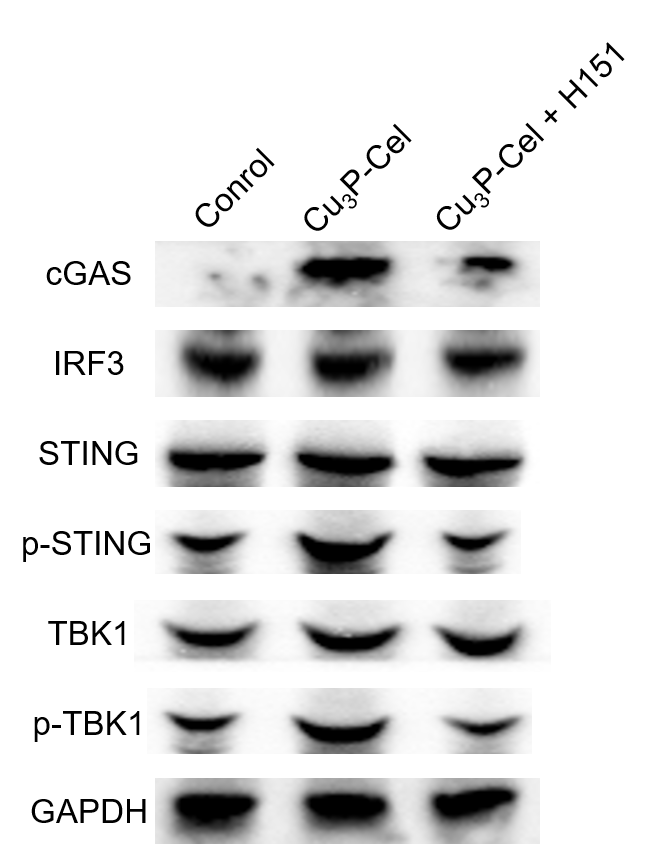


**Fig. S27.** WB analysis of cGAS, IRF3, STING, p-STING, TBK1, p-TBK1, and PD-L1 in A549 cells after different treatments.


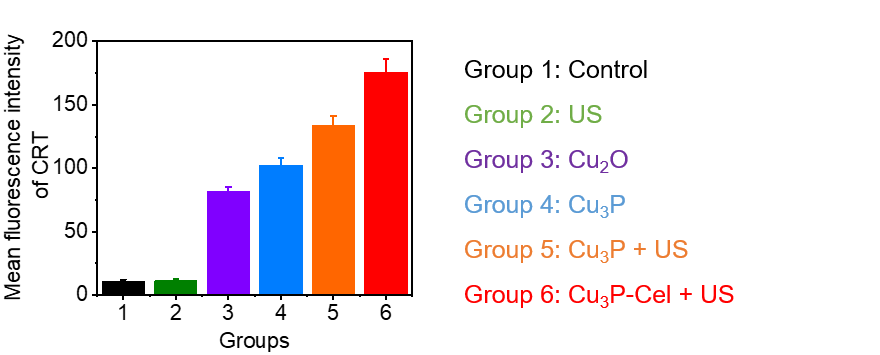


**Fig. S28.** The quantitative analysis of CRT staining in A549 cells after different treatments. Data are presented as the mean ± SD. (n = 3).


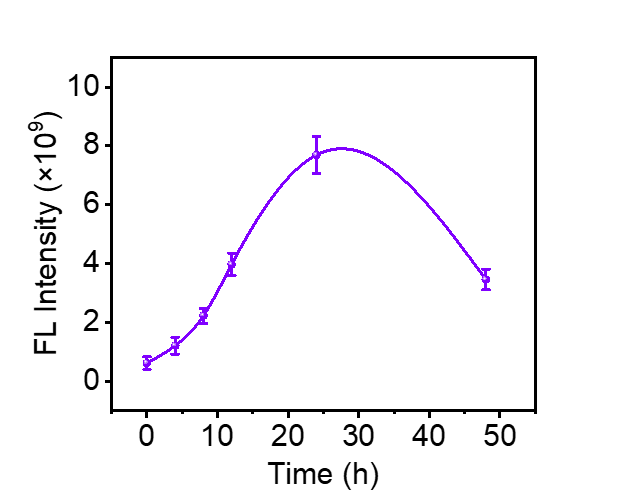


**Fig. S29.** In vivo NIR imaging of mice treated with Cu_3_P-Cel@ICG. Data are presented as the mean ± SD. (n = 3).


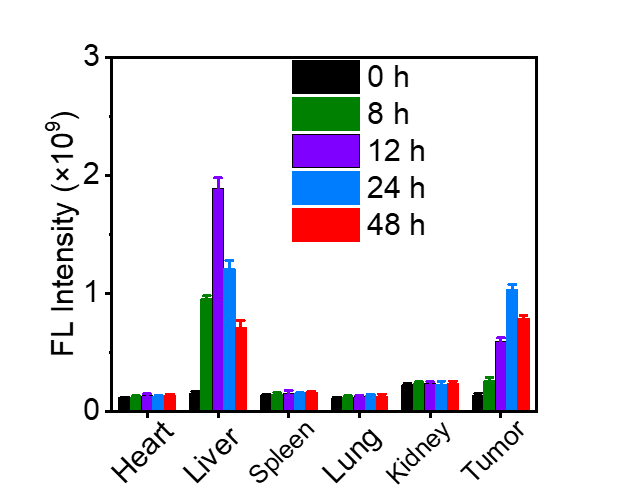


**Fig. S30.** Ex vivo NIR imaging of mice treated with Cu_3_P-Cel@ICG. Data are presented as the mean ± SD. (n = 3).


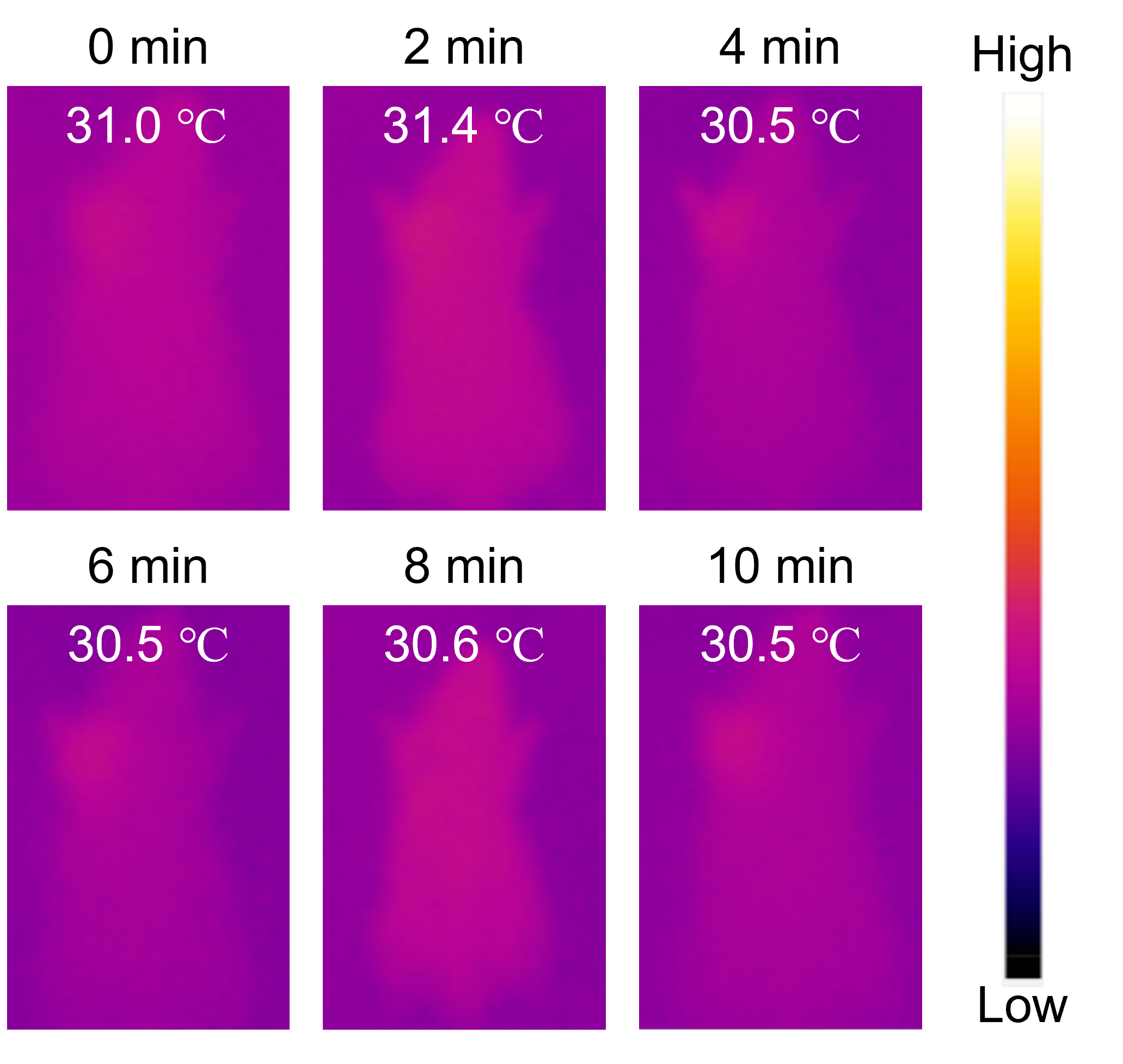


**Fig. S31.** Thermal imaging of mice during the US irradiation period.


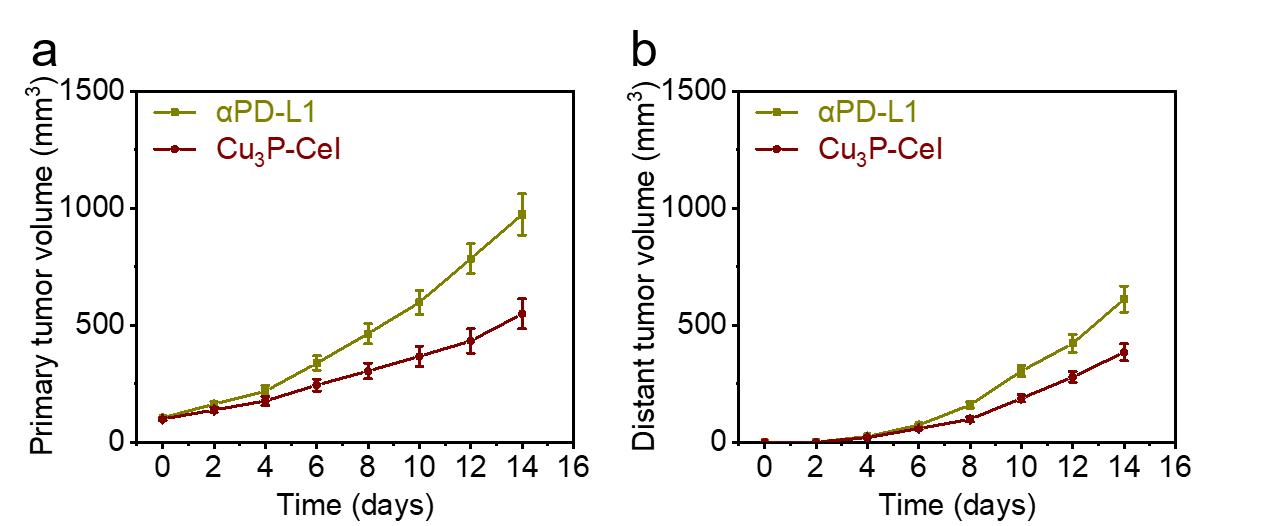


**Fig. S32.** (a, b) The antitumor effectiveness evaluation of αPD-L1 or Cu_3_P-Cel-mediated combination therapy through measuring the primary and distant tumor volume. Data are presented as the mean ± SD. (n = 5).


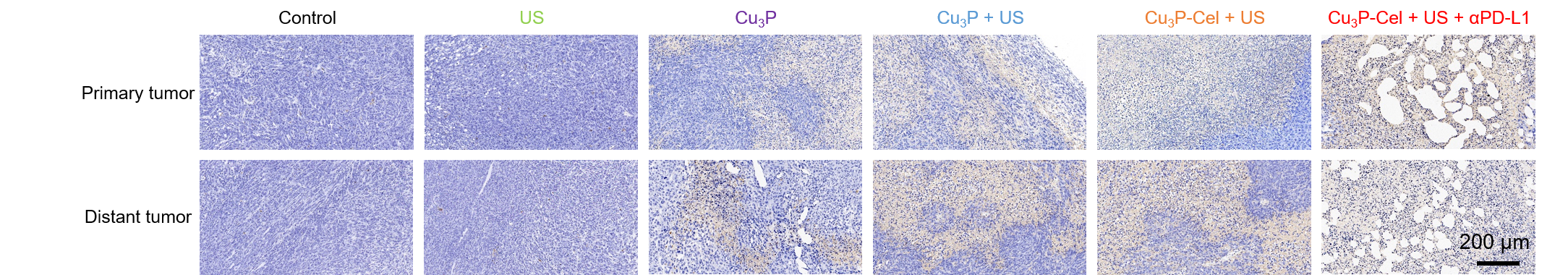


**Fig. S33.** TUNEL staining of primary and distant tumors in mice after different treatments.


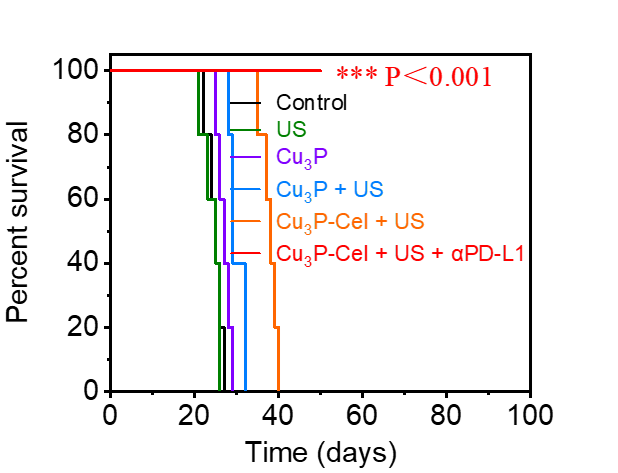


**Fig. S34.** Survival curve of mice. Data are presented as the mean ± SD. (n = 5). ***p < 0.001.


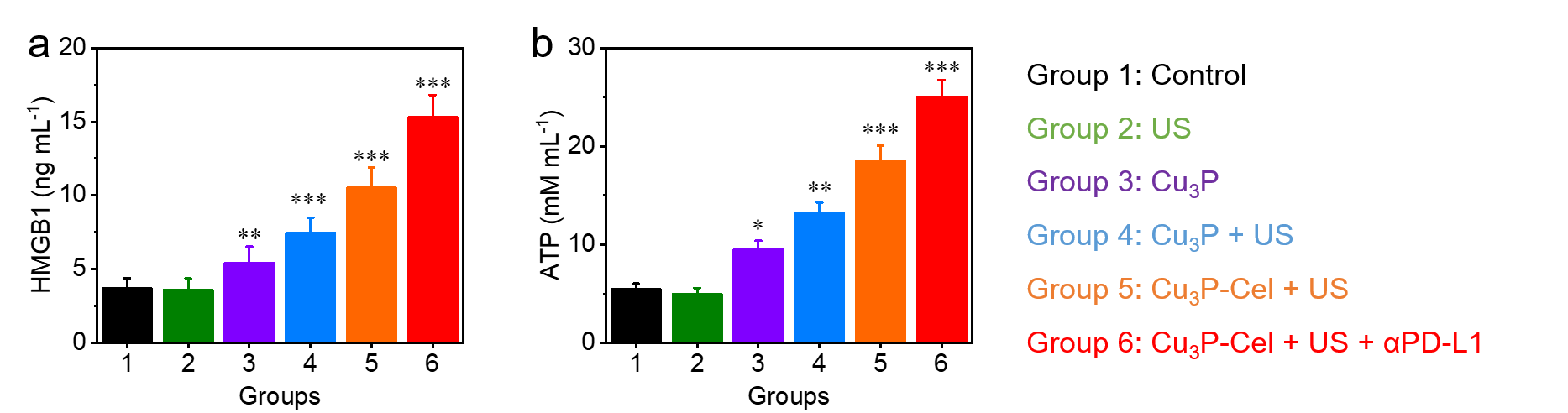


**Fig. S35.** HMGB1 and ATP levels of tumor tissues of mice after different treatments. Data are presented as the mean ± SD. (n = 3). *p < 0.05, **p < 0.01, and ***p < 0.001.


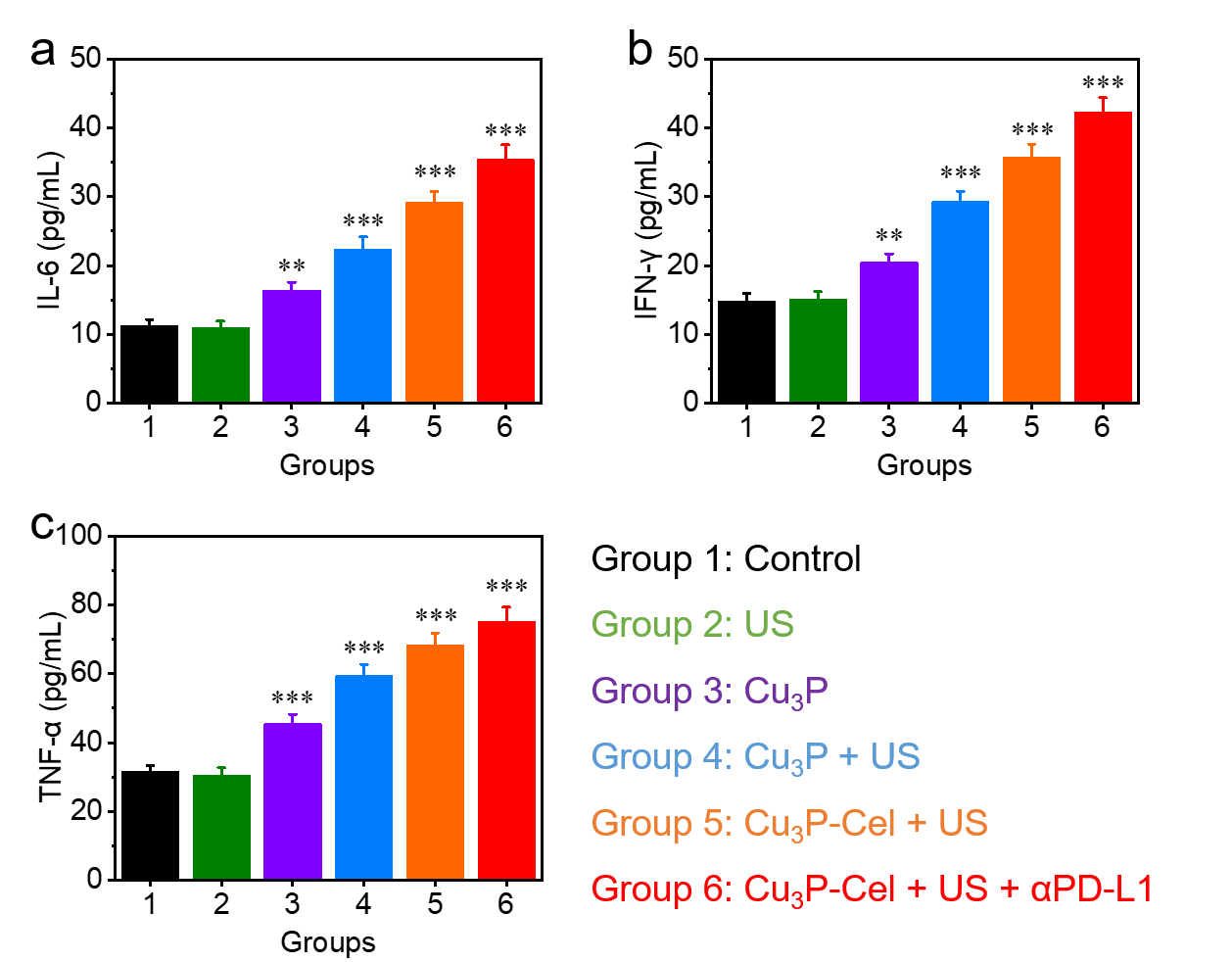


**Fig. S36.** Cytokine levels in the tumor tissues after different treatments, such as IL-6 (a), IFN-γ (b), and TNF-α (c). Data are presented as the mean ± SD. (n = 3). **p < 0.01 and ***p < 0.001.


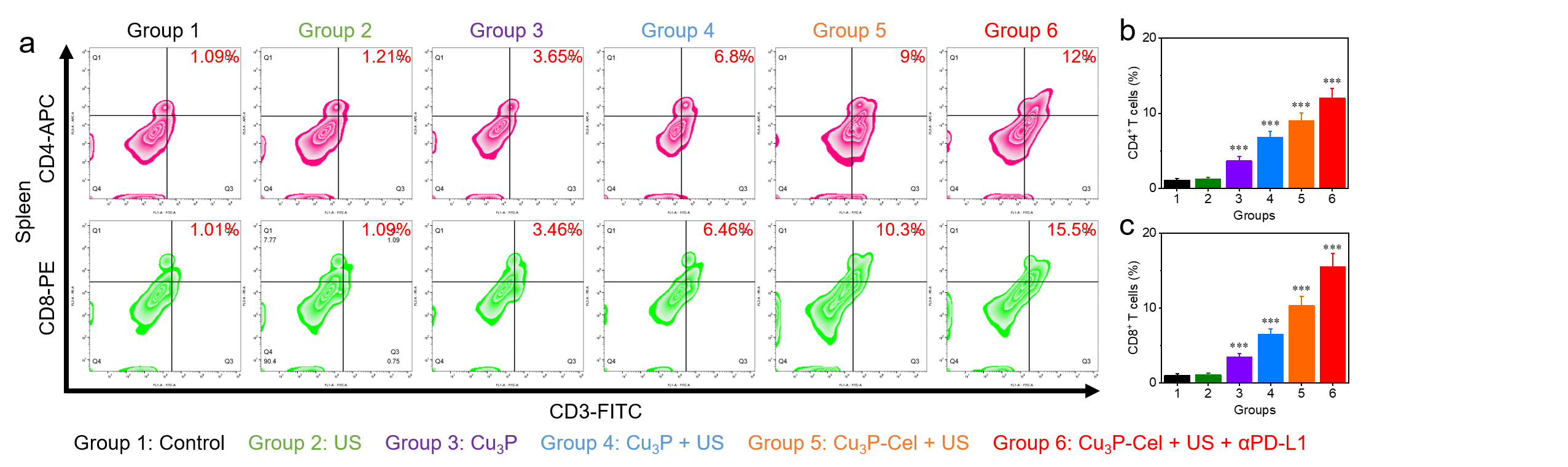


**Fig. S37.** Evaluation of the expression of CD4^+^CD8^+^ T cells in the spleen after different treatments. Data are presented as the mean ± SD. (n = 3). ***p < 0.001.


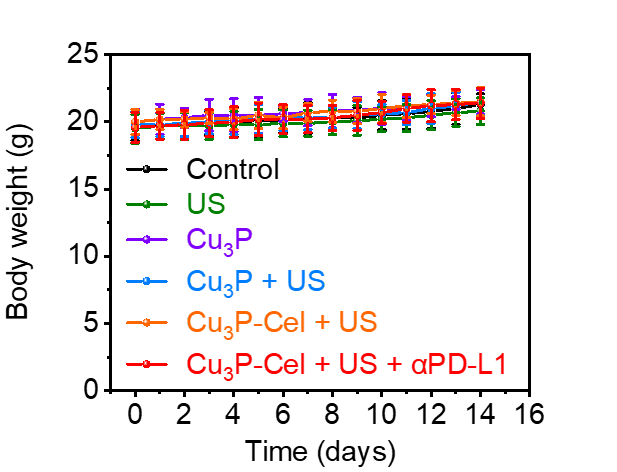


**Fig. S38.** Body weight change curve of mice. Data are presented as the mean ± SD. (n = 5).


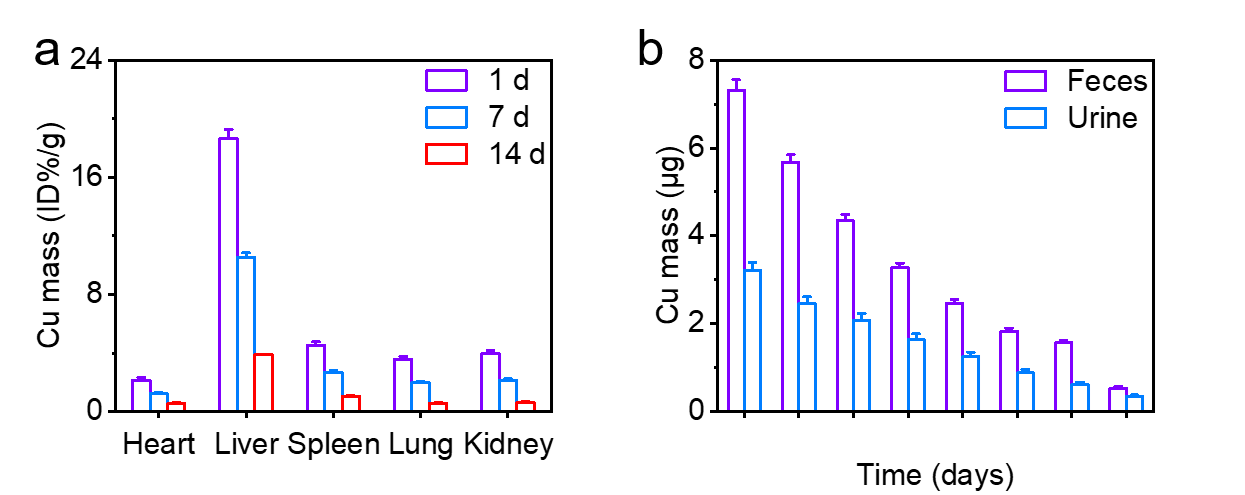


**Fig. S39.** (a) Biodistribution of Cu_3_P-Cel post i.v. injection in the major organs. (b) The detected Cu_3_P-Cel mass in urine and feces at different time points post i.v. injection. Data are presented as the mean ± SD. (n = 5).


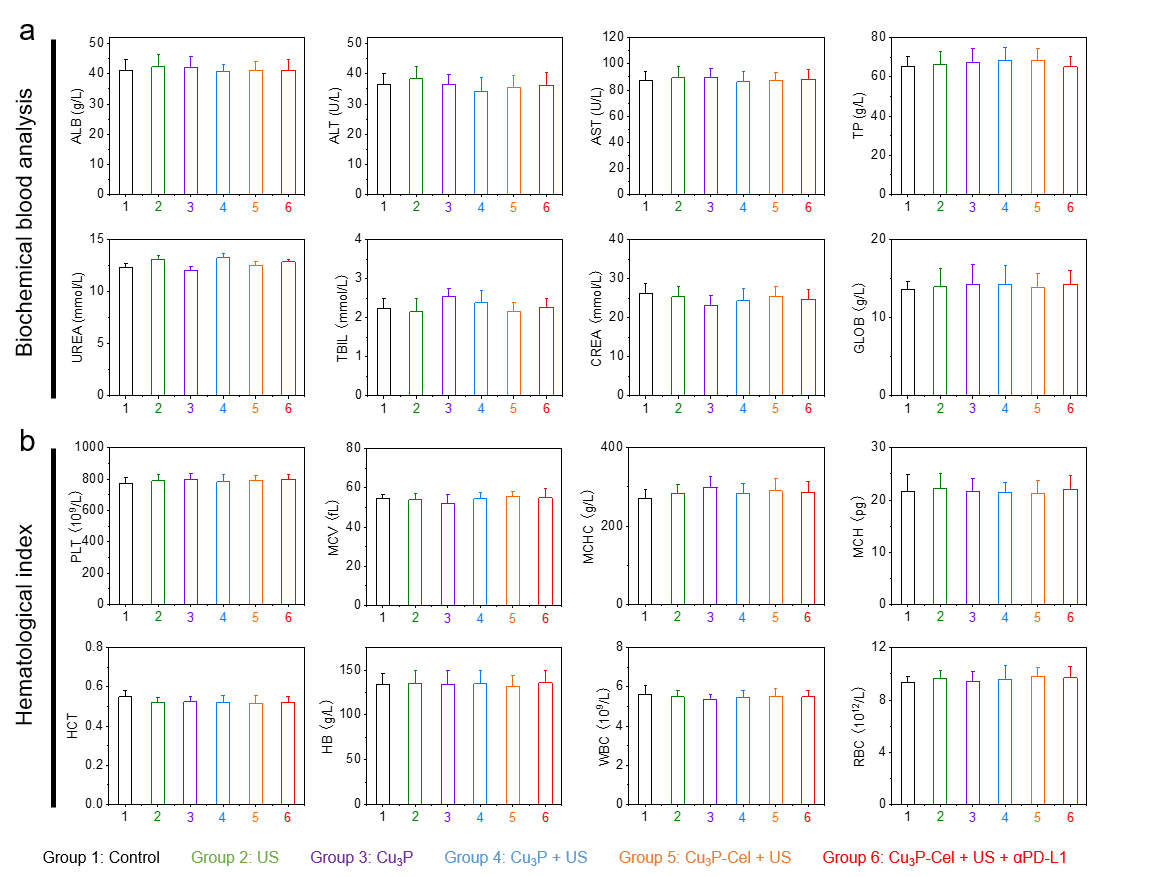


**Fig. S40.** (a-b) Biochemical blood analysis (a) and hematological index (b) of the mice that were sacrificed at 14 days after different treatments. The terms of biochemical blood analysis include ALB, ALT, AST, TP, UREA, TBIL, CREA, and GLOB. The terms of hematological index include PLT, MCV, MCHC, MCH, HCT, HB, WBC, and RBC. Data are presented as the mean ± SD. (n = 5).


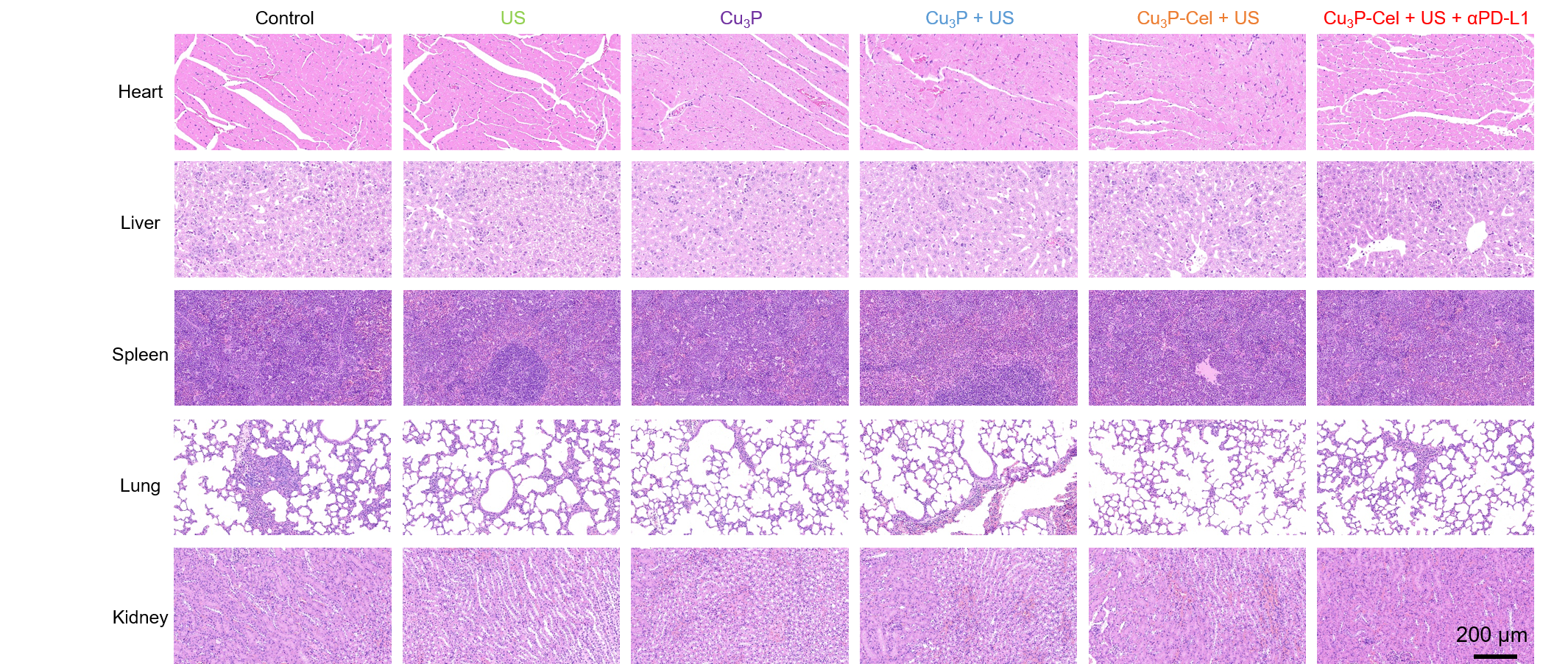


**Fig. S41.** H$\&$E staining images of the major organs in mice after different treatments.
